# Proteolytic maturation of α_2_δ represents a checkpoint for activation and neuronal trafficking of latent calcium channels

**DOI:** 10.7554/eLife.21143

**Published:** 2016-10-26

**Authors:** Ivan Kadurin, Laurent Ferron, Simon W Rothwell, James O Meyer, Leon R Douglas, Claudia S Bauer, Beatrice Lana, Wojciech Margas, Orpheas Alexopoulos, Manuela Nieto-Rostro, Wendy S Pratt, Annette C Dolphin

**Affiliations:** 1 Department of Neuroscience, Physiology and Pharmacology, University College London, London, United Kingdom; California Institute of Technology, United States

**Keywords:** Ca2+ channels, neuron, electrophysiology, Mouse, Rat

## Abstract

The auxiliary α_2_δ subunits of voltage-gated calcium channels are extracellular membrane-associated proteins, which are post-translationally cleaved into disulfide-linked polypeptides α_2_ and δ. We now show, using α_2_δ constructs containing artificial cleavage sites, that this processing is an essential step permitting voltage-dependent activation of plasma membrane N-type (Ca_V_2.2) calcium channels. Indeed, uncleaved α2δ inhibits native calcium currents in mammalian neurons. By inducing acute cell-surface proteolytic cleavage of α_2_δ, voltage-dependent activation of channels is promoted, independent from the trafficking role of α_2_δ. Uncleaved α_2_δ does not support trafficking of Ca_V_2.2 channel complexes into neuronal processes, and inhibits Ca^2+^ entry into synaptic boutons, and we can reverse this by controlled intracellular proteolytic cleavage. We propose a model whereby uncleaved α_2_δ subunits maintain immature calcium channels in an inhibited state. Proteolytic processing of α_2_δ then permits voltage-dependent activation of the channels, acting as a checkpoint allowing trafficking only of mature calcium channel complexes into neuronal processes.

**DOI:** http://dx.doi.org/10.7554/eLife.21143.001

## Introduction

The α_2_δ subunits of voltage-gated calcium channels (Ca_V_) have been identified to be key proteins in synaptic function and synaptogenesis (Dickman et al., 2008; Kurshan et al., 2009; Hoppa et al., 2012; Eroglu et al., 2009; Saheki and Bargmann, 2009). Therefore an understanding of their basic mechanism(s) of action is of paramount importance. Although Ca_V_α1 subunits form the pore and determine the main functional and pharmacological attributes of the channels (Catterall, 2000), the Ca_V_1 and Ca_V_2 channels are associated with auxiliary β and α_2_δ subunits (Flockerzi et al., 1986; Witcher et al., 1993; Takahashi et al., 1987; Liu et al., 1996). The α_2_δ subunits increase Ca_V_ currents by a mechanism that is less well understood than that of the β subunits, which have a chaperone function (Van Petegem et al., 2004; Buraei and Yang, 2010; Dolphin, 2012; Zamponi et al., 2015). The topology of the α_2_δ proteins was initially determined for skeletal muscle α_2_δ-1, but is likely to generalize for all α_2_δ subunits (Brickley et al., 1995; Gurnett et al., 1996, 1997). A single gene encodes each α_2_δ protein, which undergoes several post-translational processing steps, including proteolytic cleavage into disulfide-linked α_2_ and δ (Davies et al., 2010; Jay et al., 1991; Ellis et al., 1988; De Jongh et al., 1990). Both α_2_ and δ have been shown previously to be important for the function of α_2_δ-1 to increase Ca_V_ currents and influence the biophysical properties of the currents (Gurnett et al., 1996, 1997). The structure of the skeletal muscle Ca_V_1.1 complex has recently been determined at 3.6 Å by cryo-electron microscopy (Wu et al., 2016). It shows the interaction of the α_2_δ-1 with several extracellular linkers in Domains I-III of Ca_V_1.1. In particular the metal ion adhesion site (MIDAS) motif of the von Willebrand factor-A (VWA) domain interacts with the extracellular loop between transmembrane segments 1 and 2 in Domain I.

Regarding the mechanism of action of α_2_δ subunits, we have recently shown that α_2_δ-1 increases the density of Ca_V_2.2 channels inserted into the plasma membrane by about two-fold in undifferentiated neuro2A (N2A) cells (Cassidy et al., 2014); however the increase in currents directly attributable to α_2_δ-1 is much greater than this (Hoppa et al., 2012). In the present study, we therefore examined whether there was an additional step responsible for promoting Ca_V_2.2 calcium current function by α_2_δ subunits, in addition to their demonstrated effect on calcium channel trafficking (Cassidy et al., 2014). We have specifically tested the hypothesis that this step involves the proteolytic processing of α_2_δ-1. Our results show that proteolytic maturation of α_2_δ subunits is not only a prerequisite for voltage-dependent activation of calcium channels, but is also an essential checkpoint for trafficking of these mature calcium channels into neuronal processes.

## Results

### Mutation of six amino acids flanking the cleavage site in α_2_δ-1 prevents proteolytic processing of α_2_δ-1 into α_2_ and δ

**Figure 1.**
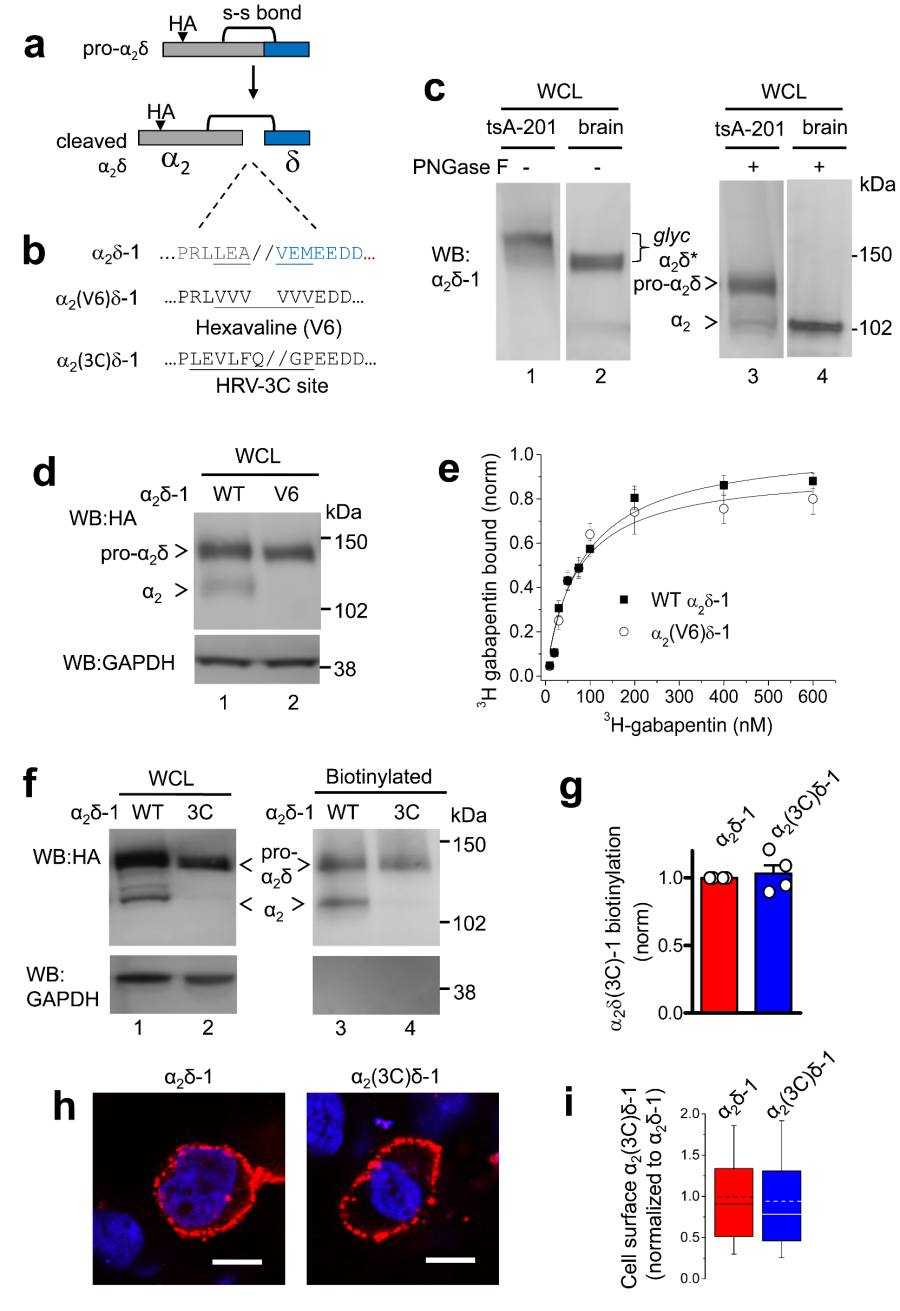
Effect of mutation of α_2_δ-1 to disrupt the proteolytic cleavage site. (**a**) Cartoon of uncleaved pro-α_2_δ-1 and cleaved α_2_δ-1, showing the approximate position of inserted HA tag and disulfide bonds between α_2_ (grey) and δ (blue). (**b**) Rat α_2_δ-1 sequence at the identified cleavage-site. The underlined sequence (including LEA//VEM) in α_2_δ-1 was mutated to a V6 or 3C-protease motif. (**c**) Comparison of glycosylated (lanes 1, 2) and deglycosylated α_2_δ-1 (lanes 3, 4), expressed in tsA-201 cells (lanes 1, 3) or present in the brain (lanes 2, 4), showing resolution between pro-α_2_δ-1 and the cleaved form of α_2_δ after deglycosylation. α_2_δ* indicates glycosylated species, and pro-α_2_δ and α_2_ indicate deglycosylated proteins. Uncleaved pro-α_2_δ-1 is observed in transfected cells, but not brain. Proteins visualized with α_2_-1 Ab. (**d**) α_2_δ-1-HA (left) and α_2_(V6)δ-1-HA (right) expressed in tsA-201 cells; proteins deglycosylated with PNGase-F. Upper panel: HA-blots, lower panel: endogenous GAPDH loading control. (**e**) Normalized binding curves, using DRM fractions from transfected tsA-201 cells, for ^3^H-gabapentin binding to WT α_2_δ-1 (■, n = 4) and α_2_(V6)δ-1 (○, n = 4). Mean (± SEM) data are fit by hyperbolae with K_D_ of 82.3 and 68.3 nM, respectively. (**f**) Imunoblot analysis of deglycosylated α_2_δ-1-HA and α_2_(3C)δ-1-HA. WCL input (lanes 1, 2) and cell-surface biotinylated material (lanes 3, 4) are shown. Upper panel: HA-blot, lower panel: endogenous GAPDH. (**g**) Mean ± SEM (and individual data points) of α_2_(3C)δ-1 (blue) cell surface levels, measured as a proportion of biotinylated: total protein, normalized to control (WT α_2_δ-1; red), for 4 experiments including that shown in (**f**). p=0.5057, one sample t test. (**h**) Cell-surface expression of α_2_δ-1-HA (left) and α_2_(3C)δ-1-HA (right) in non-permeabilized tsA-201 cells, using HA Ab (red). Nuclei visualized with DAPI (blue). Scale bars 20 μm. (**i**) Box and whisker plots for quantification of α_2_δ-1 on cell-surface, from HA fluorescence, for experiments including (**h**), for WT α_2_δ-1 (red) and α_2_(3C)δ-1 (blue). N = 290 and 317 cells, respectively, from 3 separate transfections, normalized to WT α_2_δ-1 in each experiment. p=0.259, Student’s t test. **DOI:** http://dx.doi.org/10.7554/eLife.21143.002

We identified the proteolytic cleavage site in rat α_2_δ-1 to be between A and V in the sequence LEA//VEM, by homology with the published data in rabbit (Jay et al., 1991; De Jongh et al., 1990) (Figure 1a,b). This sequence is strongly conserved in mammals (Figure 1—figure supplement 1). We initially manipulated the cleavage site in α_2_δ-1 (Figure 1b), in order to determine the extent of mutation required across this site required to completely prevent proteolytic processing. Transient expression in cell lines of wild type (WT) α_2_δ-1 resulted in only partial cleavage into α_2_ and δ subunits, clearly observed only following deglycosylation (Figure 1c, lanes 1 and 3), compared to the complete proteolytic cleavage observed in brain (Figure 1c, lanes 2 and 4). Replacing LEAVEM in α_2_δ-1 with a hexavaline (V6) sequence (Figure 1b) completely prevented its proteolytic processing, as shown by the absence of cleaved α_2_-1 on reducing gels of whole cell lysate (WCL) (Figure 1d). More conservative mutations did not effectively prevent the cleavage (data not shown). However, we found that the lack of proteolytic cleavage did not affect the ability of α_2_(V6)δ-1 to bind ^3^H-gabapentin (Figure 1e). Furthermore, the α_2_(V6)δ-1, like wild-type (WT) α_2_δ-1, was found to be concentrated in detergent-resistant membranes (DRMs; Figure 1—figure supplement 2), and the K_D_ for ^3^H-gabapentin binding in DRMs was unchanged, being 76.2 ± 7.8 nM for WT α_2_δ-1 and 85.0 ± 13.7 nM for α_2_(V6)δ-1 (Figure 1e), indicating that the uncleaved pro-form of α_2_δ-1 is likely to be correctly folded.

**Figure 1—figure supplement 1.**
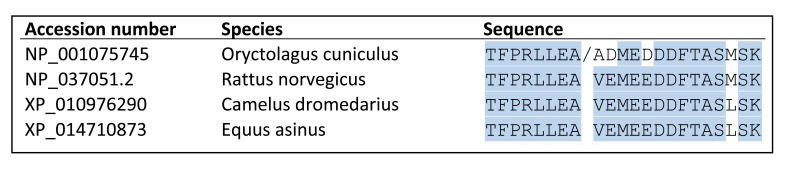
Alignment of the proteolytic cleavage site in α_2_δ-1, showing species conservation. / indicates cleavage site previously identified in rabbit α_2_δ-1 by N-terminal sequencing of delta Jay et al., 1991; De Jongh et al., 1990. **DOI:** http://dx.doi.org/10.7554/eLife.21143.003

**Figure 1—figure supplement 2.**
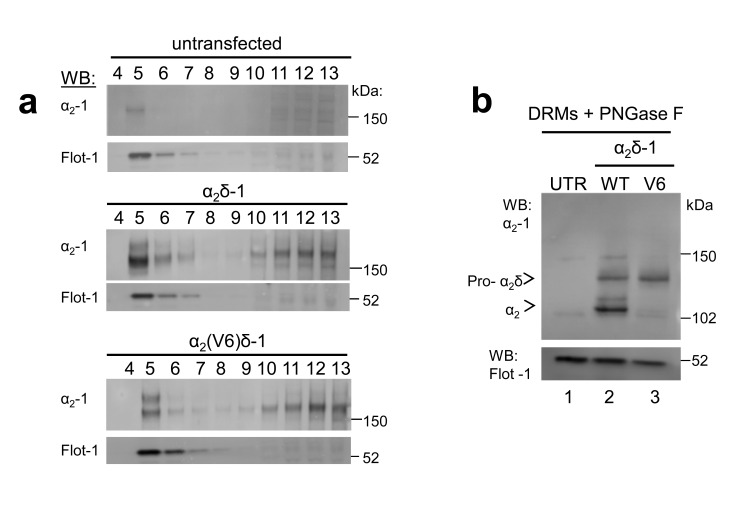
α_2_(V6)δ-1 is localised in DRMs to a similar extent to α_2_δ-1. (**a**) Sucrose gradient fractions showing a comparison of DRM localisation (lanes 2–4) of α_2_δ-1 in untransfected (UTR) tsA-201 cells (top panels, showing some endogenous α_2_δ-1), α_2_δ-1 transfected cells (middle panels) and α_2_(V6)δ-1-transfected cells (bottom panels). The upper panel is a Western blot with α_2_δ-1 mAb, and the lower panel of each set shows Flotillin-1 (Flot-1) a DRM marker. (**b**) Deglycosylation of the peak DRM fractions from the three conditions shown in A, demonstrating the absence of any proteolytic cleavage of α_2_(V6)δ-1 (lane 3). The small amount of free α_2_-1 indicated is identical to that in the UTR cells (lane 1). **DOI:** http://dx.doi.org/10.7554/eLife.21143.004

**Figure 1—figure supplement 3.**
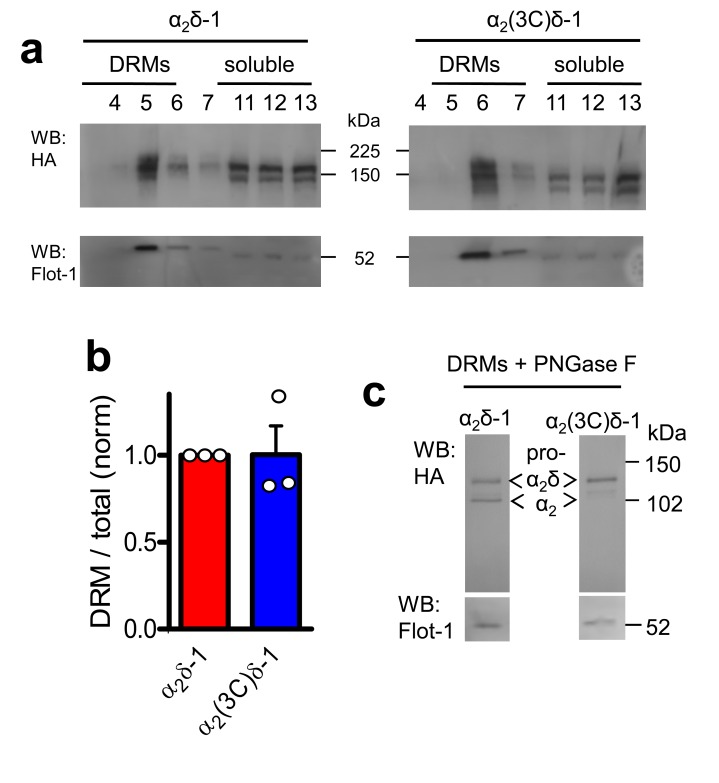
α_2_(3C)δ-1 is localised in DRMs to a similar extent to α_2_δ-1. (**a**) Comparison of DRM localisation of α_2_δ-1-HA (left panels) and α_2_(3C)δ-1-HA (right panels), isolated from transfected tsA-201 cells. Only peak DRM (4–7) and soluble (11–13) fractions are shown. The upper panel is a Western blot with HA Ab, and the lower panels show Flotillin-1 (Flot-1), a DRM marker. (**b**) Quantification of DRM localization for α_2_δ-1 (red) and α_2_(3C)δ-1 (blue), expressed as DRM/total for n = 3 experiments, normalized to the DRM localization for WT α_2_δ-1 in each experiment. Mean ± SEM and individual data points. (**c**) Deglycosylation of the peak DRM fractions from the two conditions shown in A, demonstrating the absence of any proteolytic cleavage of α_2_(3C)δ-1. Flot-1 (lower panel) is a loading control. **DOI:** http://dx.doi.org/10.7554/eLife.21143.005

### Prevention of proteolytic processing does not impair trafficking of α_2_δ-1 to the plasma membrane

We then decided to control the proteolytic processing of α_2_δ-1 by inserting a Human Rhinovirus (HRV) 3C-protease site (Cordingley et al., 1990) into α_2_δ-1, in place of the endogenous cleavage motif, to form α_2_(3C)δ-1 (Figure 1b). This substitution completely prevented its proteolytic cleavage (Figure 1f). However, α_2_(3C)δ-1 was still expressed on the cell surface (Figure 1f–i) and in DRMs (Figure 1—figure supplement 3), at similar levels to WT α_2_δ-1.

We have previously shown that the amount of proteolytically-processed α_2_δ-1 is increased on the cell surface compared to the WCL in transfected tsA-201 cells (Kadurin et al., 2012). However, the present results show that this processing of α_2_δ-1 is not essential for its trafficking to the plasma membrane.

### Prevention of proteolytic processing of α_2_δ-1 abolishes Ca_V_2.2 current enhancement

**Figure 2.**
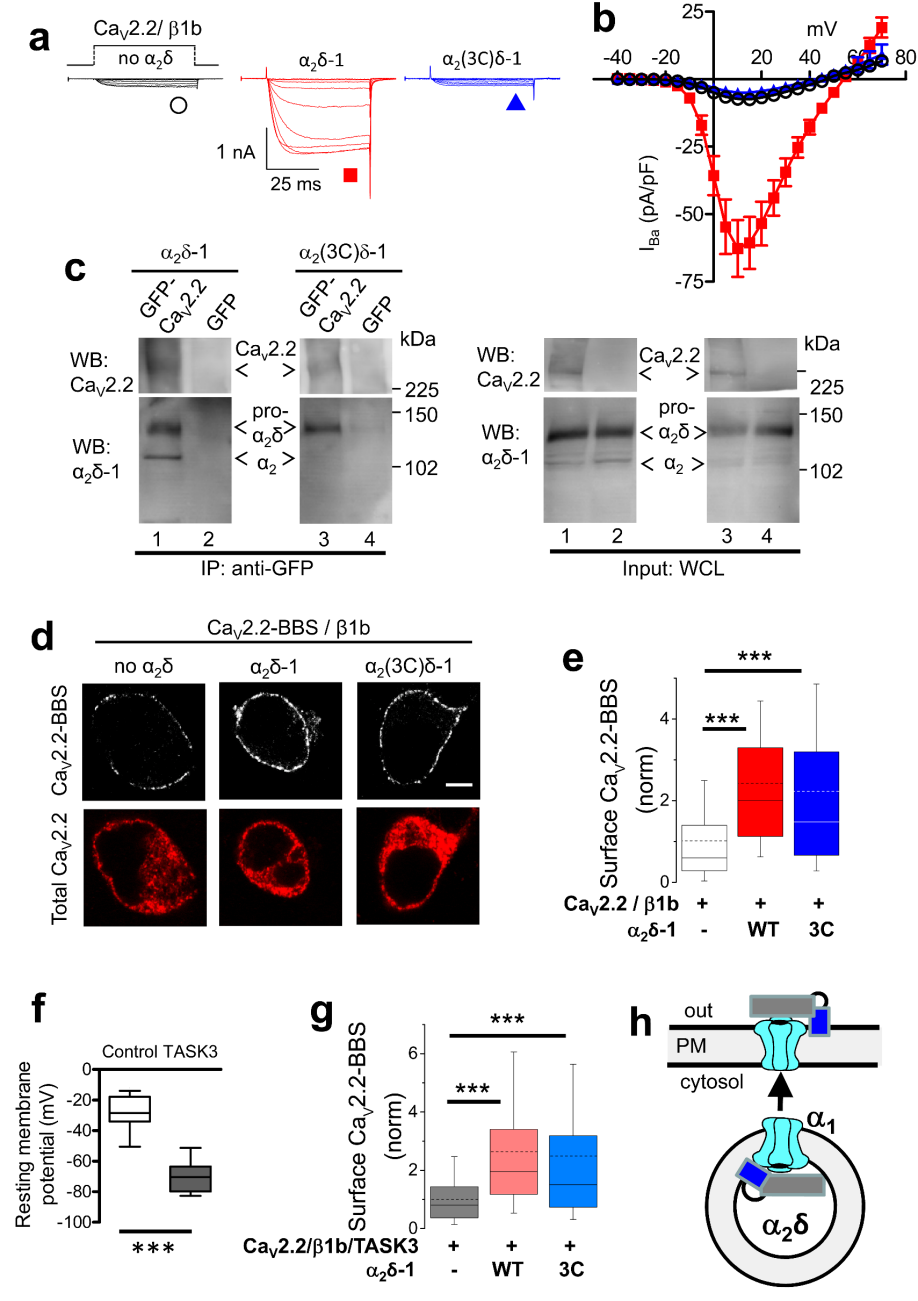
Effect of mutation of α_2_δ-1 cleavage site to an HRV-3C site on cell-surface expression and functional properties of Ca_V_2.2. (**a**) Example traces (−30 to +10 mV in 5 mV steps) for Ca_V_2.2/β1b-GFP and no α_2_δ (black traces), WT α_2_δ-1 (red traces) or α_2_(3C)δ-1 (blue traces). Charge carrier 1 mM Ba^2+^. Scale bars refer to all traces. (**b**) Mean (± SEM) *IV* curves for Ca_V_2.2/β1b-GFP and no α_2_δ (black open circles, n = 14), WT α_2_δ-1 (red squares, n = 34) or α_2_(3C)δ-1 (blue triangles, n = 21). G_max_: 0.25 ± 0.04, 1.91 ± 0.30 and 0.20 ± 0.03 nS/pF, respectively. V_50,act_: 2.85 ± 0.68, 3.33 ± 0.46 and 3.89 ± 0.53 mV, respectively. (**c**) tsA-201 cells transfected with GFP-Ca_V_2.2 (lanes 1 and 3) or GFP (lanes 2 and 4), plus β1b, and either WT α_2_δ-1 (lanes 1 and 2) or α_2_(3C)δ-1 (lanes 3 and 4). Immunoprecipitation of GFP-Ca_V_2.2 with anti-GFP Ab; WB with Ca_V_2.2 II-III loop Ab (upper panels, lanes 1 and 3) produced co-immunoprecipitation (lower panels) of WT α_2_δ-1 (lane 1) and α_2_(3C)δ-1 (lane 3), revealed by α_2_δ-1 mAb. Right panels: WCL input for lanes 1–4: upper panels, Ca_V_2.2-GFP input; lower panels, α_2_δ-1 input. All samples deglycosylated. (**d**) Immunocytochemical detection of cell-surface expression of Ca_V_2.2-BBS, with β1b, and empty vector (left), WT α_2_δ-1-HA (middle) or α_2_(3C)δ-1-HA (right) in N2A cells. Upper panel: Ca_V_2.2-BBS cell-surface staining prior to permeabilization (grey-scale); lower panel: total Ca_V_2.2 after permeabilization (II-III loop Ab, red). Scale bar 5 µm. (**e**) Quantification of Ca_V_2.2-BBS cell-surface expression (box and whisker plots) with empty vector (open bar, n = 206), WT α_2_δ-1 (red bar, n = 191) or α_2_(3C)δ-1 (blue bar, n = 181). Statistical differences: ANOVA and Bonferroni post-hoc test; ***p<0.001, compared to no α_2_δ. (**f**) Resting membrane potential of control N2A cells (white bar, n = 16) and following expression of TASK3 (gray bar, n = 12). Box and whisker plots; ***p<0.0001, Student’s t test. (**g**) Quantification of Ca_V_2.2-BBS cell-surface expression in N2A cells co-expressing TASK3, with empty vector (gray bar, n = 70), WT α_2_δ-1 (pink bar, n = 73) or α_2_(3C)δ-1 (pale blue bar, n = 81). Box and whisker plots, statistical differences: ANOVA and Bonferroni post-hoc test, compared to no α_2_δ; ***p<0.001. (**h**) Cartoon showing the ability of ‘latent’ Ca_V_2.2 (cyan) plus α_2_(3C)δ-1 (grey α_2_, blue δ), to traffic to the plasma membrane (PM). **DOI:** http://dx.doi.org/10.7554/eLife.21143.006

It has previously been established that co-expression of WT α_2_δ-1 results in an enhancement of Ca_V_2.2/β1b currents in heterologous systems (for review see [Dolphin, 2012]). We could confirm this result using β1b-GFP to ensure that all cells examined contain β1b; WT α_2_δ-1 produced an 8.6-fold increase of Ca_V_2.2/β1b currents at +10 mV (Figure 2a,b). Strikingly, no increase was observed when α_2_(3C)δ-1 was co-expressed with Ca_V_2.2/β1b (Figure 2a,b; Figure 2—figure supplement 1). This is in contrast to the ability of α_2_(3C)δ-1 to reach the plasma membrane itself. In agreement with this, another uncleavable construct used, α_2_(V6)δ-1, was also unable to increase Ca_V_2.2 currents (Figure 2—figure supplement 2).

**Figure 2—figure supplement 1.**
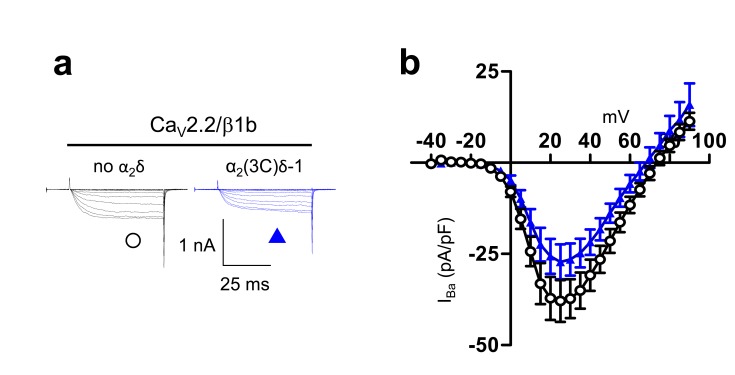
Examination of effect of α_2_(3C)δ-1 on Ca_V_2.2/β1b calcium channel currents in tsA-201 cells. These experiments were performed using 10 mM Ba^2+^ to amplify any differences in current amplitude between the two conditions examined. (**a**) Example traces (−25 to +25 mV steps) for Ca_V_2.2/β1b-GFP and either no α_2_δ (black traces) or α_2_(3C)δ-1 (blue traces). The scale bars refer to all traces. (**b**) Mean (± SEM) *IV* curves for experiments including those in (**a**), for Ca_V_2.2/β1b-GFP and either no α_2_δ (black open circles, n = 10) or α_2_(3C)δ-1-HA (blue triangles, n = 14). Peak I_Ba_ at +25 mV was −37.9 ± 5.7 pA/pF and −27.2 ± 4.8 pA/pF, respectively. G_max_ values were 0.91 ± 0.19 and 0.76 ± 0.12 nS/pF, respectively. V_50,act_ values were 13.1 ± 1.6, and 14.9 ± 1.0 mV, respectively. **DOI:** http://dx.doi.org/10.7554/eLife.21143.007

**Figure 2—figure supplement 2.**
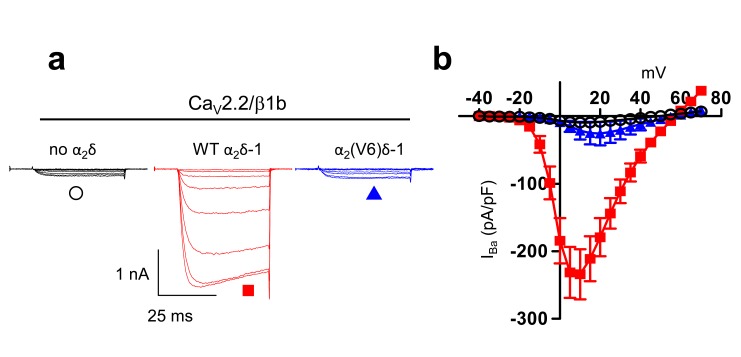
Examination of effect of α_2_(V6)δ-1 on Ca_V_2.2/β1b calcium channel currents in tsA-201 cells. (**a**) Example traces (−25 to +10 mV in 5 mV steps) for Ca_V_2.2/β1b-GFP and either no α_2_δ (black traces), α_2_δ-1 (red traces) or α_2_(V6)δ-1 (blue traces). The scale bars refer to all traces. Charge carrier 1 mM Ba^2+^ (**b**) Mean (± SEM) *IV* curves for experiments including those in (**c**), for Ca_V_2.2/β1b-GFP and either no α_2_δ (black open circles, n = 8), WT α_2_δ-1 (red squares, n = 7) or α_2_(V6)δ-1 (blue triangles, n = 8). G_max_ values were 0.32 ± 0.04, 6.72 ± 1.09 and 0.83 ± 0.52 nS/pF, respectively. V_50, act_ values were 3.6 ± 2.2,,–0.7 ± 1.1 and 5.8 ± 1.1 mV, respectively. **DOI:** http://dx.doi.org/10.7554/eLife.21143.008

**Figure 2—figure supplement 3.**
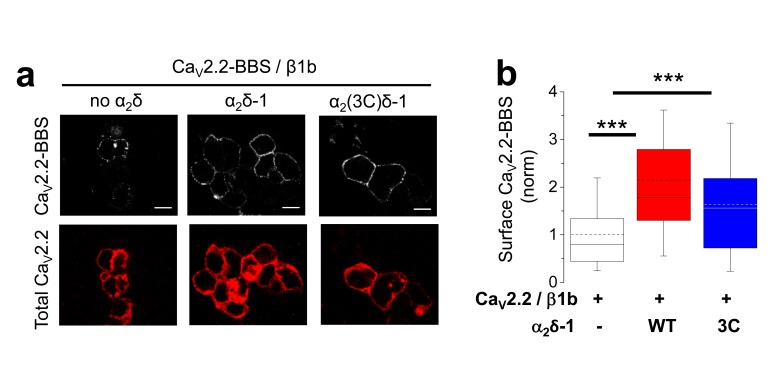
Examination of effect of α_2_(3C)δ-1 on Ca_V_2.2/β1b cell surface expression in tsA-201 cells. (**a**) Immunocytochemical detection of cell-surface expression of Ca_V_2.2-BBS, with β1b, and empty vector (left), WT α_2_δ-1-HA (middle) or α_2_(3C)δ-1-HA (right) in tsA-201 cells. Upper panel: Ca_V_2.2-BBS cell-surface staining prior to permeabilization (grey-scale); lower panel: total Ca_V_2.2 after permeabilization (II-III loop Ab, red). Scale bar 5 µm. (**b**) Quantification of Ca_V_2.2-BBS cell-surface expression in tsA-201 cells (box and 10%–90% whisker plots) with empty vector (open bar, n = 191), WT α_2_δ-1 (red bar, n = 175) or α_2_(3C)δ-1 (blue bar, n = 177). N = 3 experiments. Statistical differences: ANOVA and Bonferroni post-hoc test; ***p<0.001, compared to no α_2_δ. **DOI:** http://dx.doi.org/10.7554/eLife.21143.009

### Proteolytic processing of α_2_δ-1 is not required for the interaction with Ca_V_2.2, and trafficking of the complex to the plasma membrane in cell lines

We next examined whether α_2_(3C)δ-1 is impaired in its interaction with Ca_V_2.2, which would explain its inability to increase Ca_V_2.2 currents. However, we found that Ca_V_2.2 co-immunoprecipitates with α_2_(3C)δ-1 to the same extent as WT α_2_δ-1 (Figure 2c). Furthermore, using extracellularly-tagged Ca_V_2.2 to quantify the channels inserted into the plasma membrane (Cassidy et al., 2014), we found that in undifferentiated N2A cells, the uncleaved α_2_(3C)δ-1 remains capable of increasing the cell surface density of Ca_V_2.2 channels (123% increase compared to Ca_V_2.2/β1b alone), by a similar extent to WT α_2_δ-1 (140% increase; Figure 2d,e). α_2_(3C)δ-1 also increased cell surface expression of Ca_V_2.2 in the tsA-201 cells used for electrophysiology (Figure 2—figure supplement 3). We then asked whether the depolarized resting membrane potential of N2A cells might influence their ability to traffic Ca_V_2.2. We therefore co-expressed the leak K^+^ channel, TASK3 (Kim et al., 2000), to hyperpolarize their membrane potential (Figure 2f), in order to determine whether this would differentially affect Ca_V_2.2 trafficking supported by WT α_2_δ-1 and α_2_(3C)δ-1. This was not the case, as a similar increase in Ca_V_2.2 surface expression was observed in the presence of TASK3 for Ca_V_2.2, with either WT α_2_δ-1 or α_2_(3C)δ-1 (Figure 2g).

Thus, when heterologously expressed in cell lines, α_2_(3C)δ-1 cause an increase of Ca_V_2.2 surface expression, but does not result in an increase of Ca_V_2.2 currents (see cartoon in Figure 2h), strongly suggesting α_2_(3C)δ-1 may play an inhibitory role on Ca_V_2.2 function.

### Proteolytic processing of α_2_δ-1 is essential for voltage-dependent activation of Ca_V_2.2 Currents

**Figure 3.**
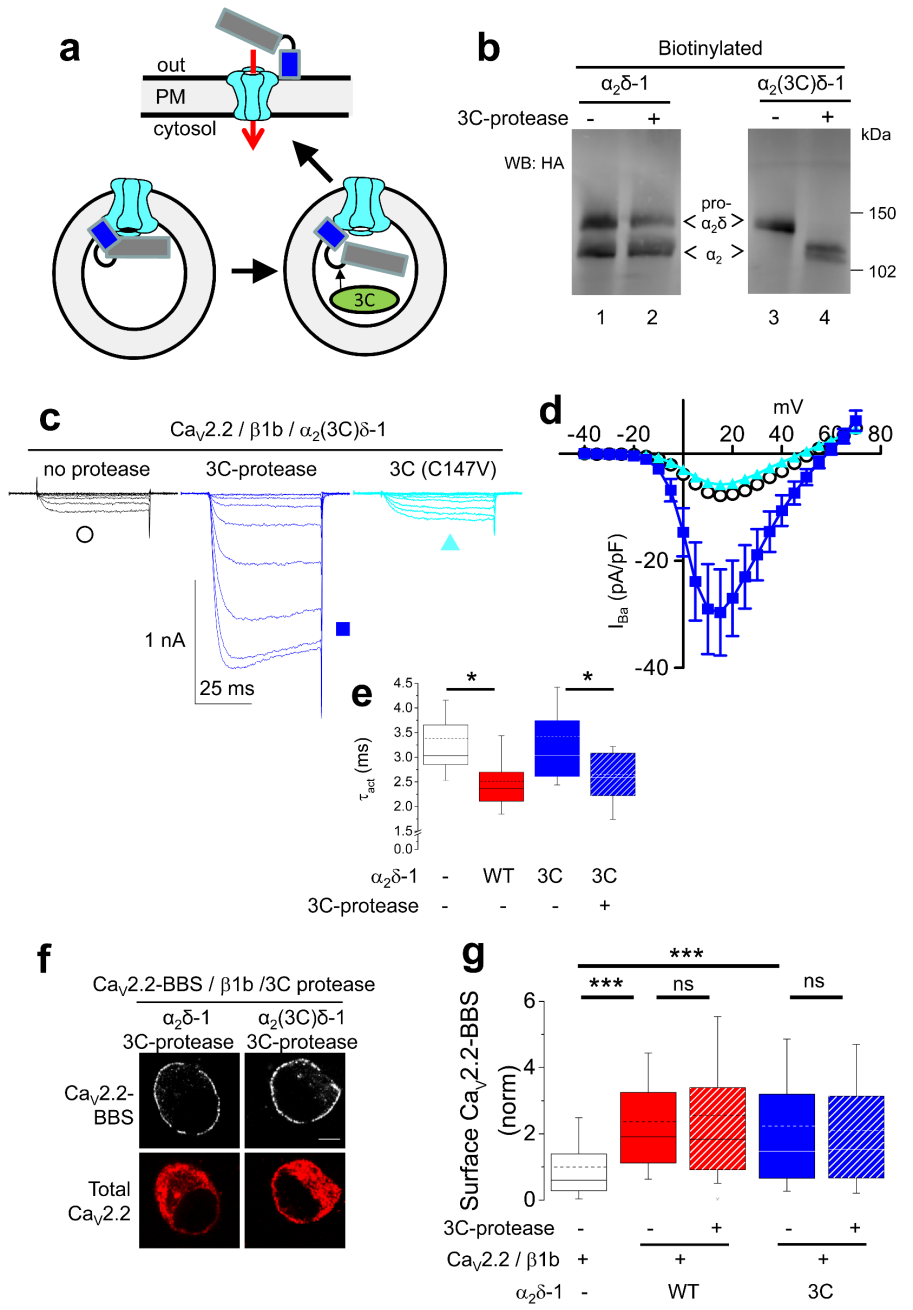
Effect of proteolytic cleavage of α_2_δ-1 containing an HRV-3C cleavage site on cell-surface expression and functional properties of Ca_V_2.2. (**a**) Cartoon showing intracellular cleavage by 3C-protease (green) of α_2_(3C)δ-1 (gray/blue) associated with Ca_V_2.2 (cyan). (**b**) Deglycosylated, cell-surface biotinylated fractions for α_2_δ-1-HA (lanes 1 and 2) and α_2_(3C)δ-1-HA (lanes 3 and 4), expressed in tsA-201 cells without (lanes 1 and 3) or with (lanes 2 and 4) 3C-protease. Representative of n = 4 experiments. WCL in Figure 3—figure supplement 1 (**c**) Example traces (−30 to +10 mV steps) for Ca_V_2.2/β1b-GFP/α_2_(3C)δ-1-HA and no protease (black traces), 3C-protease (blue traces) or inactive mutant 3C-(C147V) protease (cyan traces). Charge carrier 1 mM Ba^2+^. Scale bars refer to all traces. (**d**) Mean (± SEM) *IV* curves for Ca_V_2.2/β1b-GFP/α_2_(3C)δ-1-HA and no protease (black open circles, n = 26), 3C-protease (blue squares, n = 22) or 3C-(C147V)-protease (cyan triangles, n = 23). G_max_: 0.26 ± 0.04, 0.90 ± 0.22 and 0.22 ± 0.03 nS/pF, respectively. G_max_ values in the presence of the active 3C-protease were greater than in the absence of protease or presence of 3C-(C147V)-protease (Kruskal-Wallis test with Dunn’s multiple comparison post-hoc test, p<0.05). V_50,act_: 6.05 ± 0.82, 5.18 ± 0.74 and 6.20 ± 1.20 mV, respectively. (**e**) Time constant of activation (τ_act_) for I_Ba_ at +10 mV for Ca_V_2.2/β1b-GFP with no α_2_δ-1 (open bar, n = 14), WT α_2_δ-1-HA (red bar, n = 25), α_2_(3C)δ-1-HA (blue bar, n = 21) or α_2_(3C)δ-1-HA + 3C-protease (blue hatched bar, n = 17). Box and whisker plots. Statistical significance determined by ANOVA and Bonferroni’s post-hoc test (*p<0.05). (**f**) Immunocytochemical detection of cell-surface Ca_V_2.2-BBS, plus β1b, and α_2_δ-1-HA (left panel) or α_2_(3C)δ-1-HA (right panel), with 3C-protease. Upper panel: Ca_V_2.2-BBS cell-surface staining (grey-scale), lower panel total Ca_V_2.2 (II-III loop staining). Scale bar 5 µm. (**g**) Lack of effect of 3C-protease (hatched bars) on cell-surface expression of Ca_V_2.2-BBS following expression of Ca_V_2.2-BBS/β1b in N2A cells, with no α_2_δ (open bar, n = 206), WT α_2_δ-1 (red bar, n = 212), WT α_2_δ-1 and 3C-protease (red hatched bar, n = 192), α_2_(3C)δ-1 (blue bar, n = 181) or α_2_(3C)δ-1 and 3C-protease (blue hatched bar, n = 200). Box and whisker plots. Statistical differences determined by ANOVA and Bonferroni’s post-hoc test (***p<0.001; ns: p>0.05). **DOI:** http://dx.doi.org/10.7554/eLife.21143.010

In order to determine whether α_2_(3C)δ-1 could be cleaved at the inserted 3C-protease site, we then co-expressed an ER-lumen-targeted 3C-protease together with α_2_(3C)δ-1 (Figure 3a). This resulted in efficient proteolytic cleavage of α_2_(3C)δ-1 into α_2_ and δ, as shown by the appearance of a band of the size of the α_2_ moiety, which reached the cell surface (Figure 3b; Figure 3—figure supplement 1a). As expected, co-expression of an inactive form of 3C-protease with a mutation in the catalytic site (C147V) failed to affect α_2_(3C)δ-1 (Figure 3—figure supplement 1b).

**Figure 3—figure supplement 1.**
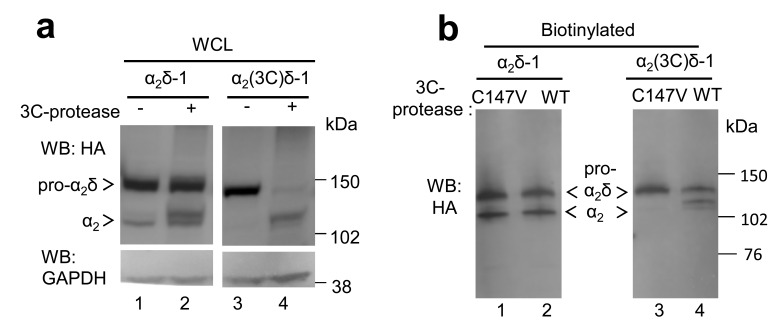
The effect of 3C-protease on α_2_δ-1 and α_2_(3C)δ-1 expressed in tsA-201 cells. (**a**) Deglycosylated WCL for the experiment shown in Figure 3b; α_2_δ-1-HA (lanes 1 and 2) and α_2_(3C)δ-1-HA (lanes 3 and 4), expressed in tsA-201 cells, without (lanes 1 and 3) or with (lanes 2 and 4) 3C-protease. Upper panel shows HA blot, lower panel: shows GAPDH blot loading control. (**b**) Cell surface biotinylation for α_2_δ-1-HA (lanes 1 and 2) and α_2_(3C)δ-1-HA (lanes 3 and 4), expressed in tsA-201 cells with inactive C147V (lanes 1 and 3) or (lanes 2 and 4) 3C-protease. Proteins were deglycosylated. WB was performed with HA Ab. **DOI:** http://dx.doi.org/10.7554/eLife.21143.011

**Figure 3—figure supplement 2.**
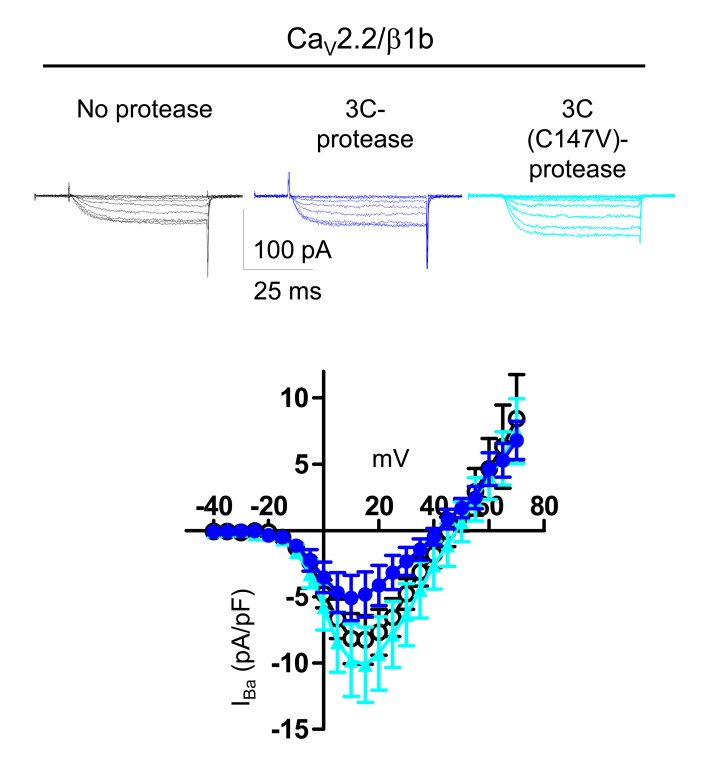
Lack of effect of 3C-protease on Ca_V_2.2/β1b currents expressed in tsA-201 cells. Top: example traces (−25 to +15 mV steps) for Ca_V_2.2/β1b-GFP and either no protease (black traces), WT 3C-protease (blue traces) or inactive mutant 3C-protease (C147V, cyan traces). The charge carrier was 1 mM Ba^2+^. The scale bars refer to all traces. Bottom: Mean (± SEM) *IV* curves for experiments including those shown, for Ca_V_2.2/β1b-GFP and either no protease (black open circles, n = 9), WT 3C-protease (blue circles, n = 11) or inactive mutant 3C-protease (C147V) (cyan triangles, n = 9). Peak I_Ba_ at +15 mV was −8.2 ± 1.8 pA/pF, −4.8 ± 1.7 pA/pF and −10.1 ± 2.8 pA/pF, respectively. G_max_ values were 0.32 ± 0.08, 0.20 ± 0.06 and 0.36 ± 0.09 nS/pF, respectively. V_50, act_ values were 4.1 ± 0.7, 1.2 ± 1.2 and 3.8 ± 1.0 mV, respectively. **DOI:** http://dx.doi.org/10.7554/eLife.21143.012

Next we addressed the important question of whether proteolytic cleavage by 3C-protease could restore the currents through Ca_V_2.2 channels containing α_2_(3C)δ-1 subunits. In order to do this, we co-expressed 3C-protease with Ca_V_2.2/β1b/α_2_(3C)δ-1, and found that α_2_δ-1-mediated Ca_V_2.2 current enhancement was robustly rescued by the active protease, whereas the inactive mutant 3C-protease (C147V) had no effect (Figure 3c–e). This was not accompanied by any change in Ca_V_2.2 trafficking (Figure 3f,g). The peak I_Ba_ increase due to the 3C-protease was 5.4-fold at +10 mV, compared to inactive protease (Figure 3c,d). Co-expression of 3C-protease with α_2_(3C)δ-1 also resulted in an increased activation rate of the Ca_V_2.2 currents, to the same extent as WT α_2_δ-1 (Figure 3e). As expected, when α_2_(3C)δ-1 was not present, there was no increase in Ca_V_2.2 currents attributable to 3C-protease (Figure 3—figure supplement 2).

In summary, expression of active, but not the inactive form of 3C-protease results in cleavage of α_2_(3C)δ-1 and rescues enhancement of Ca_V_2.2 currents by α_2_(3C)δ-1, without any effect on trafficking, providing evidence that proteolytic processing of α_2_δ-1 is required to promote voltage-dependent activation Ca_V_2.2 channels.

### Replacement of the endogenous proteolytic site in WT α_2_δ-3 with a 3C-protease site allows controlled processing of α_2_δ-3 to rescue Ca_V_2.2 currents

**Figure 4.**
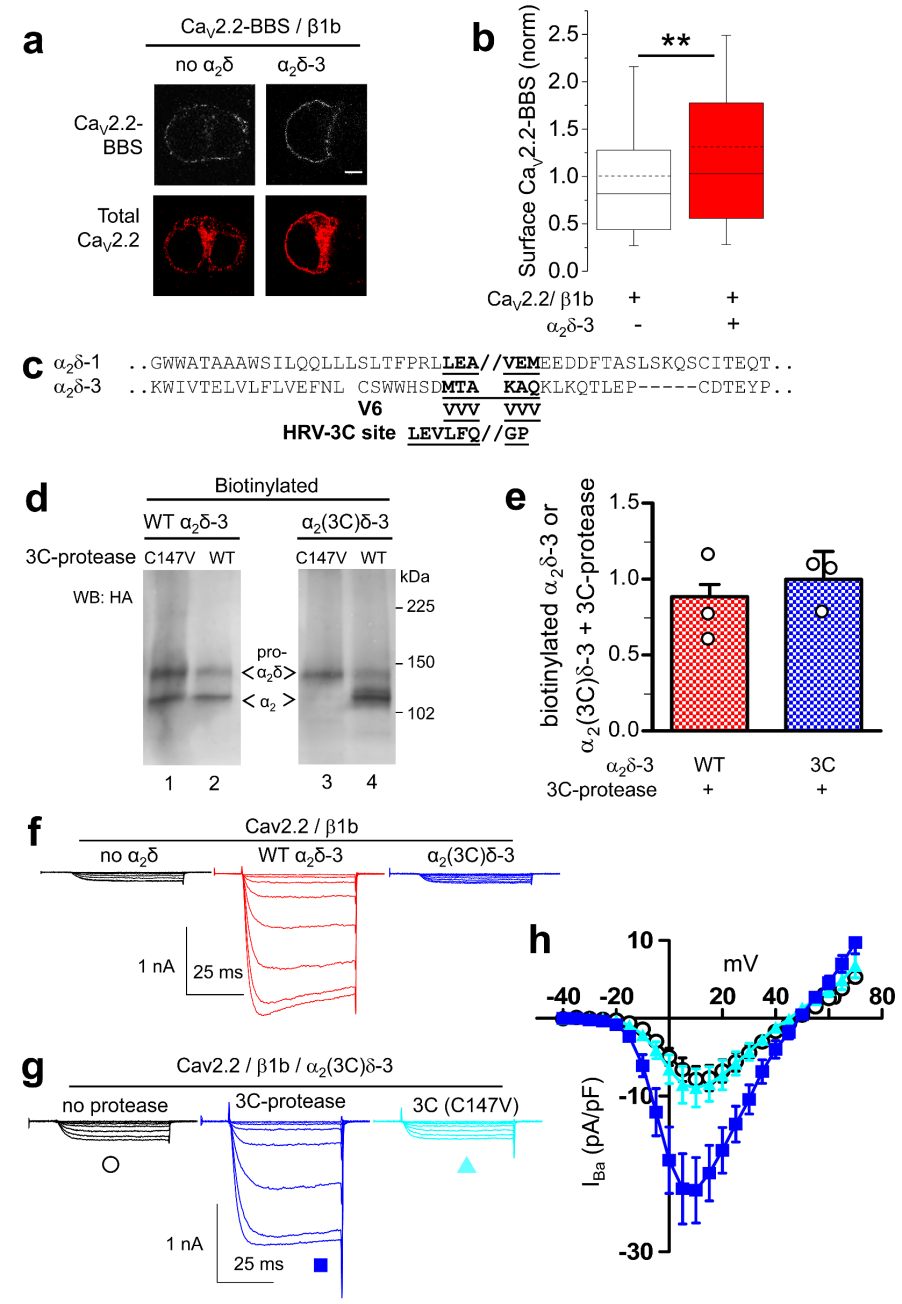
Effect of proteolytic cleavage of α_2_δ-3 containing an HRV-3C cleavage site on cell-surface expression and functional properties of Ca_V_2.2. (**a**) Images showing cell-surface Ca_V_2.2-BBS (upper row, white), and total Ca_V_2.2 (II-III loop Ab, lower row, red), for Ca_V_2.2-BBS/β1b in N2A cells, with empty vector (panel 1) or α_2_δ-3-HA (panel 2). Scale bar 5 µm. (**b**) Quantification (box and whisker plots) of effect α_2_δ-3 on cell-surface Ca_V_2.2-BBS following expression of Ca_V_2.2-BBS/β1b with empty vector (open bar, n = 188) or WT α_2_δ-3 (red bar, n = 188). Statistical difference determined by Student’s t test, **p=0.0028. (**c**) Alignment of α_2_δ-3 sequence around the predicted cleavage site with α_2_δ-1, showing weak homology. Underlined sequence (MTAKAQ) mutated to V6 or HRV-3C cleavage motif. (**d**) α_2_δ-3-HA (lanes 1, 2) and α_2_(3C)δ-3-HA (lanes 3, 4) expressed in tsA-201 cells, with either inactive (C147V, lanes 1, 3) or WT 3C-protease (WT, lanes 2, 4), cell-surface biotinylated and deglycosylated. Full WB and corresponding WCL in Figure 4—figure supplement 3 (**e**) Quantification of cell-surface expression of WT α_2_δ-3-HA (red speckled bar) and α_2_(3C)δ-3-HA (blue speckled bar), with 3C-protease, normalized relative to inactive 3C-protease (C147V) for n = 3 experiments. Data are mean (± SEM) and individual data points: p=0.4721 for WT α_2_δ-3-HA and p=0.9513 for α_2_(3C)δ-3 (1 sample t-test compared to respective control). (**f**) Example traces (−30 to +5 mV steps) for Ca_V_2.2/β1b-GFP with no α_2_δ (black traces), WT α_2_δ-3 (red traces) or α_2_(3C)δ-3 (blue traces). G_max_: 0.24 ± 0.03, 1.46 ± 0.22 and 0.21 ± 0.03 nS/pF respectively. V_50,act_: 0.91 ± 1.015, 1.01 ± 0.85 and 4.03 ± 1.04 mV, respectively. (**g**) Example traces (−30 to +10 mV steps) for Ca_V_2.2/β1b-GFP/α_2_(3C)δ-3 and no protease (black traces), 3C-protease (blue traces) or inactive 3C-protease (C147V) (cyan traces). For (**f**) and (**g**), charge carrier 1 mM Ba^2+^, scale bars refer to all traces. (**h**) Mean (± SEM) *IV* curves for Ca_V_2.2/β1b-GFP/α_2_(3C)δ-3 without protease (black open circles, n = 28), with 3C-protease (blue squares, n = 29) or inactive 3C-(C147V)-protease (cyan triangles, n = 24). G_max_: 0.28 ± 0.05, 0.70 ± 0.11 and 0.30 ± 0.08 nS/pF, respectively. G_max_ for 3C-protease condition larger than other two conditions (Kruskal-Wallis test with Dunn’s post-hoc test, p<0.05). V_50, act_: 4.0 ± 0.7, 0.3 ± 0.7 and 1.5 ± 0.6 mV, respectively. **DOI:** http://dx.doi.org/10.7554/eLife.21143.013

In order to further distinguish between the effects of α_2_δ subunits on Ca_V_2.2 channel trafficking and voltage-dependent activation, we also examined the behavior of α_2_δ-3, because we surmised it might have a less prominent trafficking effect, as it contains an incomplete MIDAS motif (Whittaker and Hynes, 2002) in its VWA domain, which we have shown is essential for the trafficking and function of α_2_δ-1 and α_2_δ-2 (Hoppa et al., 2012; Cassidy et al., 2014; Canti et al., 2005). Indeed, we found that α_2_δ-3 produced a much smaller increase than α_2_δ-1 (31%, compared to ~140%) on Ca_V_2.2 cell surface expression (Figure 4a,b), which we attribute to the absence of a complete MIDAS motif in α_2_δ-3 (Whittaker and Hynes, 2002).

**Figure 4—figure supplement 1.**
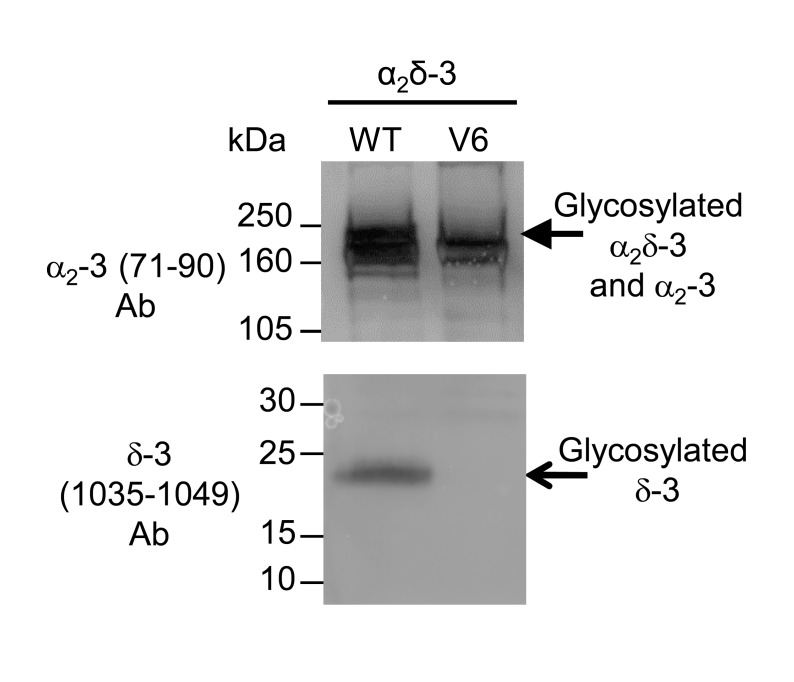
Lack of cleavage of α_2_(V6)δ-3. The expression of α_2_δ-3 compared to α_2_(V6)δ-3. The constructs were expressed transiently in tsA-201 cells; peak lipid raft fractions were taken for western blotting. There was a complete loss of free δ-3 (lower panel) in α_2_(V6)δ-3 (lane 2), compared to WT α_2_δ-3 (lane 1). α_2_δ-3 is shown in the upper panel. **DOI:** http://dx.doi.org/10.7554/eLife.21143.014

**Figure 4—figure supplement 2.**
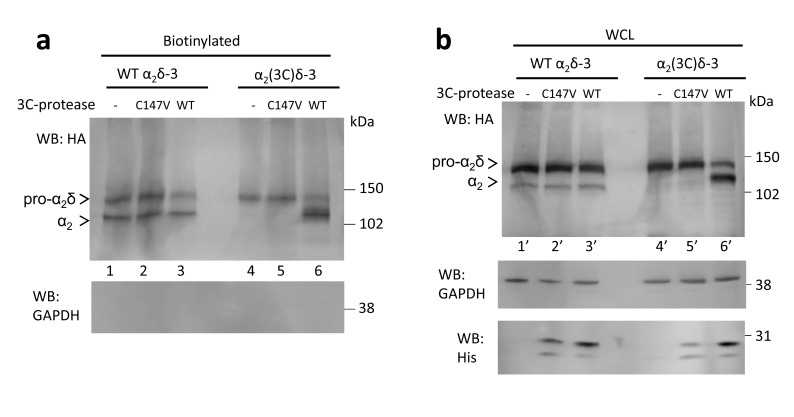
Effect of 3C-protease on expression and cleavage of α_2_(3C)δ-3. Full data from experiment shown in Figure 4d. WT α_2_δ-3-HA (lanes 1, 2 and 3) and α_2_(3C)δ-3-HA (lanes 4, 5 and 6) were expressed in tsA-201 cells, either without or with inactive (C147V, lanes 2 and 5) or WT 3C-protease (WT, lanes 3 and 6), subjected to cell surface biotinylation and streptavidin pull-down. Proteins were deglycosylated with PNGase-F. (**a**) shows biotinylated proteins and (**b**) shows WCL. The upper blot with HA Ab, shows that WT, but not inactive mutant, 3C-protease cleaved α_2_(3C)δ-3 (lane 6). The lower blot shows GAPDH, indicating that no intracellular proteins were biotinylated, and the bottom blot in (**b**) shows expression of the His-tagged 3C-proteases. **DOI:** http://dx.doi.org/10.7554/eLife.21143.015

**Figure 4—figure supplement 3.**
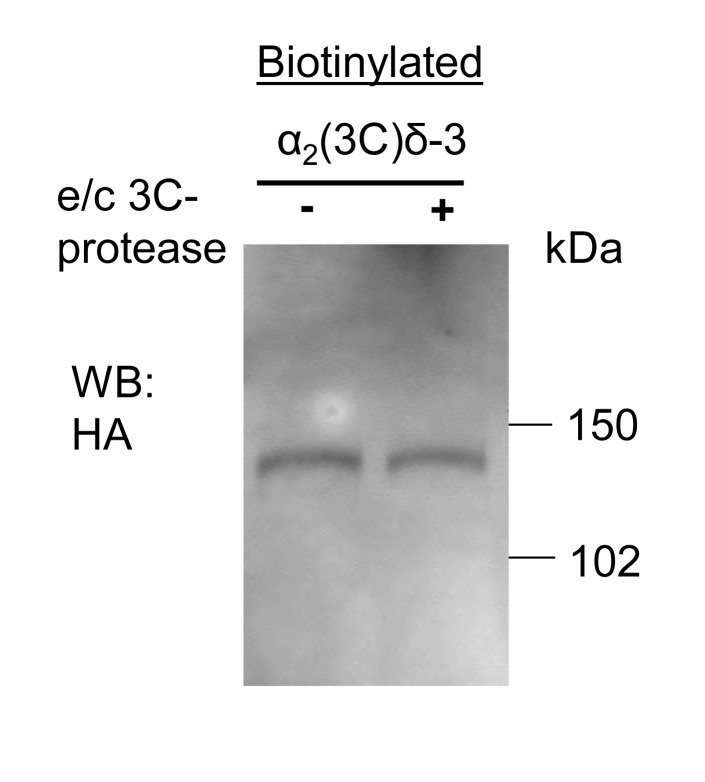
Lack of effect of purified 3C-protease on expression and cleavage of α_2_(3C)δ-3. Incubation of cells expressing α_2_(3C)δ-3-HA with 3C-protease enzyme did not result in cleavage on the cell surface. Cell surface biotinylated deglycosylated α_2_(3C)δ-3-HA, from cells incubated without (left) or with (right) 3C-protease. **DOI:** http://dx.doi.org/10.7554/eLife.21143.016

The site of proteolytic cleavage in α_2_δ-3 has not been previously determined, although it is fully cleaved into α_2_ and δ in vivo (Davies et al., 2010), and enhances Ca_V_2.2 currents (Davies et al., 2010). We therefore first identified a potential cleavage site in α_2_δ-3 by sequence alignment (Figure 4c), and then showed by mutational analysis that a V6 mutation across the predicted cleavage site prevented proteolytic cleavage, determined by the appearance of the δ-3 moiety in a reducing gel (Figure 4—figure supplement 1), and also prevented the functional effects of α_2_δ-3 on Ca_V_2.2 currents (data not shown). We then replaced this sequence in α_2_δ-3 with the HRV-3C motif, to form α_2_(3C)δ-3 (Figure 4c). We found that α_2_(3C)δ-3 was expressed on the cell surface to a similar extent to WT α_2_δ-3 (Figure 4d), despite the complete absence of proteolytic cleavage (Figure 4d, lane 3). Importantly, when the 3C-protease was co-expressed, we found that α_2_(3C)δ-3 was still present on the cell surface, and was almost completely cleaved at the inserted HRV-3C site (Figure 4d, lane 4), although this had no effect on WT α_2_δ-3 (Figure 4d,e; Figure 4—figure supplement 2a,b). As we also found for α_2_(3C)δ-1, α_2_(3C)δ-3 did not increase Ca_V_2.2 currents, whereas WT α_2_δ-3 produced a 6.6-fold increase (Figure 4f). However, inducing proteolytic cleavage of α_2_(3C)δ-3 by 3C-protease substantially rescued the enhancement of Ca_V_2.2 currents, whereas the mutant protease 3C (C147V) did not (Figure 4g,h). The increase in peak I_Ba_ due to the 3C protease was 2.7-fold at +10 mV, compared to inactive protease.

In summary WT α_2_δ-3 has a much smaller effect on Ca_V_2.2 cell surface trafficking than WT α_2_δ-1, but proteolytic cleavage of α_2_(3C)δ-3 still produces a substantial increase in Ca_V_2.2 currents.

### Proteolytic processing of α_2_δ subunits on the cell surface leads to rescue of Ca_V_2.2 Currents

**Figure 5.**
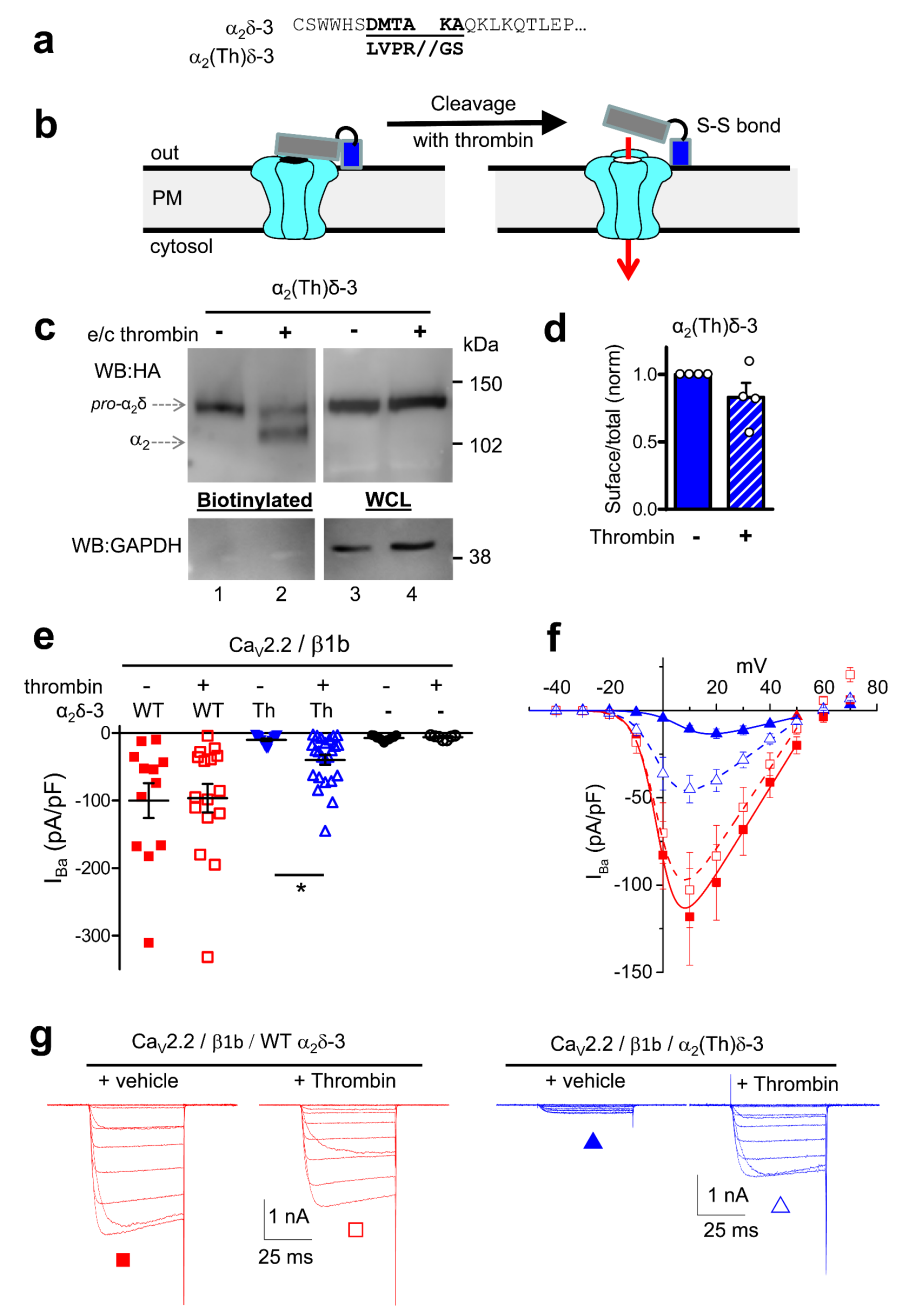
Effect of thrombin on the properties and function of α_2_δ-3 containing a thrombin proteolytic cleavage site. (**a**) Sequence at α_2_δ-3 cleavage site mutated to a thrombin cleavage site. (**b**) Cartoon of thrombin cleavage of α_2_δ-3 on cell-surface. (**c**) Cell-surface biotinylation (left panel) shows efficient cleavage of cell-surface α_2_(Th)δ-3-HA (lane 2), with no effect on total α_2_(Th)δ-3-HA in WCL (right panel, lane 4). Samples were deglycosylated prior to loading. (**d**) Quantification of cell surface biotinylation experiments such as those shown in (**c**), indicating that thrombin does not decrease the amount of α_2_(Th)δ-3-HA on the cell surface (hatched blue bar), normalized to vehicle application in each experiment (solid blue bar). Mean (± SEM) and individual data points for n = 4; p=0.2105, 1 sample t test. (**e**) Mean (± SEM) and individual data points of peak I_Ba_ at +10 mV, for Ca_V_2.2/β1b with WT α_2_δ-3 (red squares), α_2_(Th)δ-3 (blue triangles) or no α_2_δ (black circles) and either no protease (closed symbols), or 60 min thrombin incubation (open symbols). The charge carrier was 2 mM Ba^2+^. For data without or with thrombin, respectively, n = 12, 16 for WT α_2_δ-3; 15, 24 for α_2_(Th)δ-3 and 11, 7 without α_2_δ, from at least 3 independent transfections. Statistical difference between thrombin and vehicle determined by Kruskal-Wallis ANOVA and Dunn’s multiple comparison post-hoc test, *p<0.05. (**f**) Mean (± SEM) full *IV* curves for the same conditions as in (**e**) (excluding the no α_2_δ data), fitted with a modified Boltzmann equation to +50 mV. G_max_ values (nS/pF) were 2.80 ± 0.61 (WT α_2_δ-3; n = 10), 2.60 ± 0.55 (WT α_2_δ-3 plus thrombin, n = 15), 0.43 ± 0.08 (α_2_(Th)δ-3; n = 14), 1.26 ± 0.21 (α_2_(Th)δ-3 plus thrombin, n = 21). V_50,act_ values (mV) were +0.44 ± 1.71 (WT α_2_δ-3), + 0.55 ± 1.28 (WT α_2_δ-3 plus thrombin), +9.34 ± 0.08 (α_2_(Th)δ-3), +0.87 ± 1.38 (α_2_(Th)δ-3 plus thrombin). (**g**) Example Ba^2+^ currents (from −50 to +60 mV) for the four conditions shown in (**f**). **DOI:** http://dx.doi.org/10.7554/eLife.21143.017

In order to further probe the role of proteolytic processing of the α_2_δ subunits on calcium channel currents, independent of their trafficking effects, we next turned to application of extracellular protease. However, we found that incubation of cells expressing α_2_(3C)δ-1 or α_2_(3C)δ-3 with purified 3C-protease did not result in their proteolytic cleavage at the plasma membrane (Figure 4—figure supplement 3, and data not shown). As an alternative approach, we inserted a thrombin cleavage site into α_2_δ-3 to produce α_2_(Th)δ-3 (Figure 5a,b). Importantly, we first showed that extracellular thrombin did not cleave WT α_2_δ-3 (Figure 5—figure supplement 1), and we found that α_2_(Th)δ-3 reached the cell surface as an uncleaved protein (Figure 5c,d; Figure 5—figure supplement 1). Furthermore, application of thrombin in the extracellular medium then produced marked cleavage of α_2_(Th)δ-3 on the cell surface (Figure 5c), and the optimal incubation period was about 60 min (Figure 5—figure supplement 1). This experiment was not attempted for α_2_δ-1 as preliminary studies revealed that α_2_δ-1 contained an ectopic thrombin cleavage site (data not shown).

**Figure 5—figure supplement 1.**
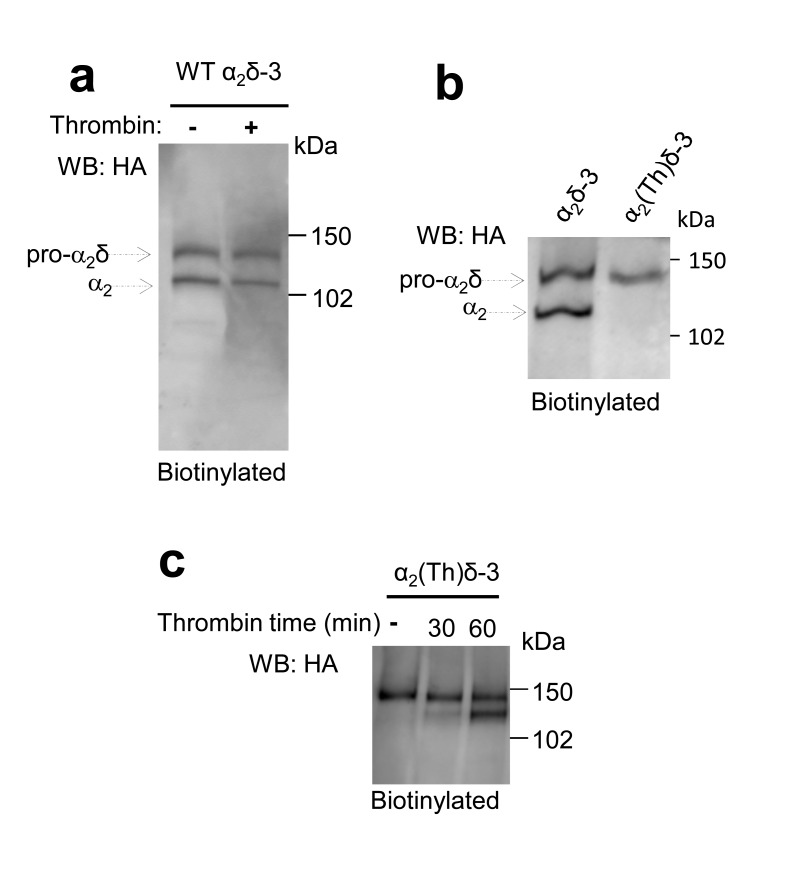
Controls for cleavage of α_2_(Th)δ-3 by thrombin. (**a**) Deglycosylated cell surface biotinylation for tsA-201 cells expressing WT α_2_δ-3-HA and incubated either with vehicle or thrombin for 60 min (lanes 1 and 2), showing WT α_2_δ-3 is not cleaved by thrombin. (**b**) Deglycosylated cell surface biotinylation for tsA-201 cells expressing WT α_2_δ-3-HA or α_2_(Th)δ-3-HA (lanes 1 and 2), showing α_2_(Th)δ-3 reaches cell surface. (**c**) Deglycosylated cell surface biotinylation for tsA-201 cells expressing α_2_(Th)δ-3-HA and incubated with thrombin for 0, 30 and 60 min (lanes 1–3), showing α_2_(Th)δ-3 is cleaved on cell surface at 60 min. **DOI:** http://dx.doi.org/10.7554/eLife.21143.018

In line with previous results, uncleaved α_2_(Th)δ-3 failed to mediate Ca_V_2.2 current enhancement (Figure 5e). However, when thrombin was added to cells expressing Ca_V_2.2/β1b and α_2_(Th)δ-3, prior to Ba^2+^ current recording, it resulted in an acute increase in I_Ba_ amplitude (Figure 5e–g), clearly demonstrating that cleavage of α_2_(Th)δ-3 has a direct effect to rescue Ca_V_2.2/β1b/α_2_(Th)δ-3 currents. In contrast, extracellular application of thrombin had no effect on Ca_V_2.2/β1b/WT α_2_δ-3 or Ca_V_2.2/β1b currents (Figure 5e–g). Therefore, the functional role of α_2_δ-3 on Ca_V_2.2 channels can be acutely restored at the plasma membrane via specific proteolytic cleavage of extracellular α_2_(Th)δ-3.

### Identification of the subcellular location of physiological cleavage of α_2_δ-1 subunits in DRG neurons

**Figure 6.**
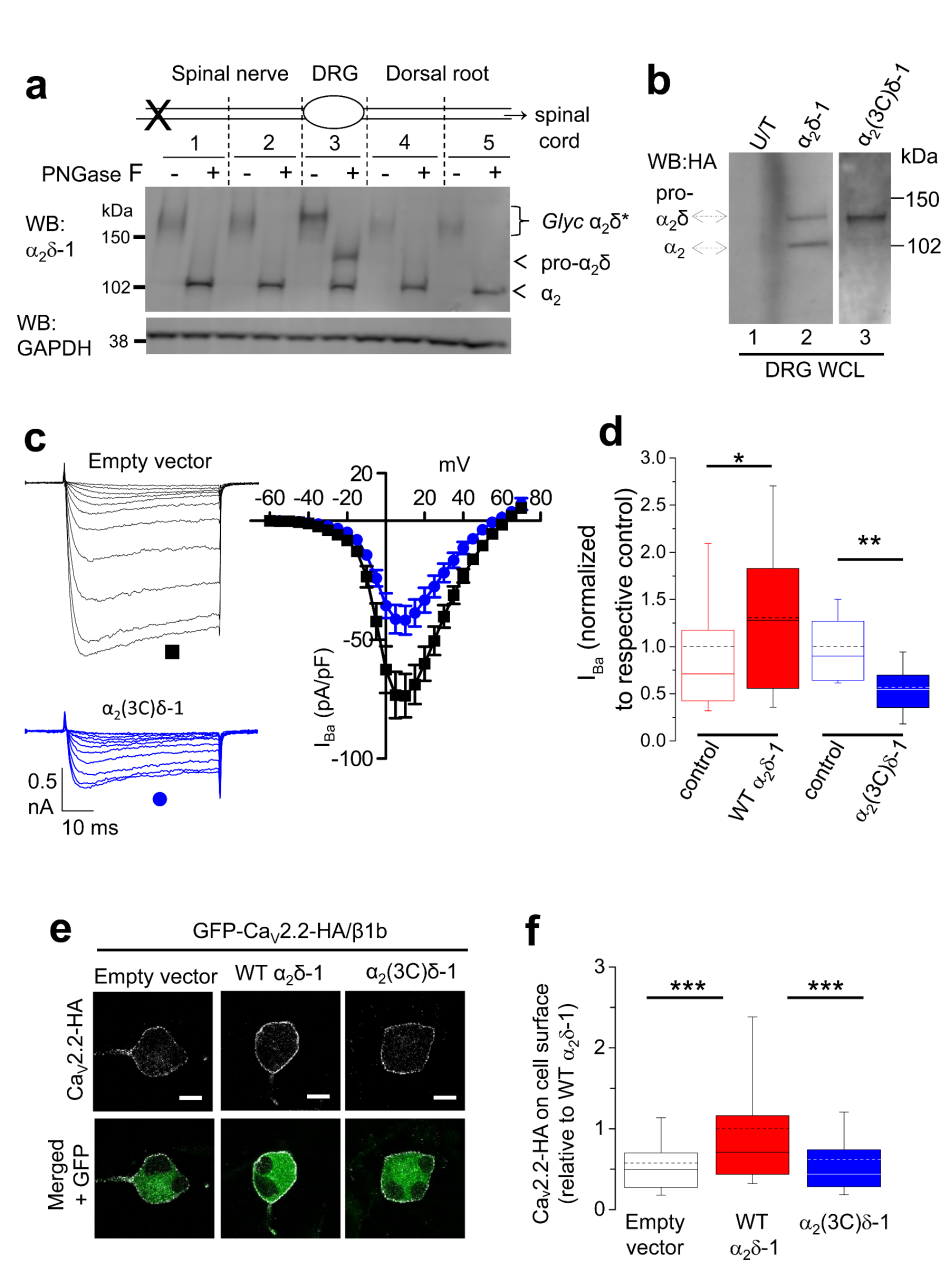
Proteolytic processing of endogenous α_2_δ-1 and effect of exogenous expression of α_2_δ-1 and α_2_(3C)δ-1 in DRGs. (**a**) DRGs, spinal nerves and dorsal roots from rats, 4 days after SNL, were dissected and segmented according to the diagram. X marks site of ligation. Tissue was pooled from 4 rats. It was either treated or not with PNGase-F as indicated, and reduced with DTT; deglycosylation allows resolution of two α_2_-immunoreactive bands. Unprocessed α_2_δ-1 is present only in the cell body compartment (segment 3) and is distinct from processed α_2_-1 (indicated by arrows). Lower blot is GAPDH loading control. (**b**) WCL for empty vector-transfected DRGs (U/T, lane 1); WT α_2_δ-1-HA-transfected DRGs (lane 2); α_2_(3C)δ-1-HA transfected DRGs (lane 3). WB: anti-HA. (**c**) Left: Example traces (−45 to +5 mV steps) for control (empty vector-transfected) DRG neurons (black traces, top) and DRGs transfected with α_2_(3C)δ-1 (blue traces, bottom). The charge carrier is 1 mM Ba^2+^. The scale bars refer to all traces. Right: Mean (± SEM) *IV* curves for control DRG neurons (black squares, n = 12) and DRGs transfected with α_2_(3C)δ-1 (blue circles, n = 14), from 3 independent experiments. G_max_ values were 2.20 ± 0.30 and 1.26 ± 0.14 nS/pF, respectively; p=0.0061 (Student’s t test). V_50, act_ values were −1.5 ± 1.2 and −1.5 ± 0.7 mV, respectively. (**d**) Comparison of normalized peak I_Ba_ in control DRGs (open red bar, n = 55) and WT α_2_δ-1-transfected DRGs (closed red bar, n = 54) including data from Figure 2d in, or comparison of control DRGs (open blue bar, n = 12) with α_2_(3C)δ-1 transfected DRGs (closed blue bar, n = 14). Box and whisker plots. Statistical differences, Student’s t test: *, p=0.048, **p=0.0067 compared to respective control. (**e**) Confocal optical sections (1 µm) showing GFP-Ca_V_2.2-HA in non-permeabilized DRG neurons (top, white), when co-transfected with β1b and either empty vector (left), WT α_2_δ-1 (middle) or α_2_(3C)δ-1 (right). GFP fluorescence is shown in the merged lower panel. Scale bars: 10 µm. (**f**) Box and whisker plot of cell surface HA fluorescence density as a ratio of internal GFP density for Ca_V_2.2-HA expression in DRG somata, transfected with empty vector (open bar, n = 81), WT α_2_δ-1 (red bar, n = 133) or α_2_(3C)δ-1 (blue bar, n = 159). ***p<0.001, 1 way ANOVA and Bonferroni post hoc test. **DOI:** http://dx.doi.org/10.7554/eLife.21143.019

The α_2_δ-1 protein is extensively up-regulated in DRG neurons, following experimental peripheral nerve injury, such as spinal nerve ligation (SNL), and is trafficked to presynaptic terminals along the axon in intracellular trafficking vesicles (Bauer et al., 2009). This up-regulation allowed us to obtain sufficient protein to follow the processing of α_2_δ-1. Following dissection of DRGs and associated axons, we observed that α_2_δ-1 was fully glycosylated in the cell bodies of the DRGs, but remained only partially cleaved into α_2_ and δ (Figure 6a, tissue segment 3). We have shown previously by electron microscopy that within the cell body, α_2_δ-1 is present both in the endoplasmic reticulum and at the plasma membrane, whereas in axons it is entirely associated with intracellular transport vesicles (Bauer et al., 2009). In the axons, we observed that proteolytic cleavage of α_2_δ-1 was complete, both in the spinal nerve (Figure 6a, tissue segments 1, 2), and in the dorsal roots (Figure 6a, tissue segments 4, 5). This finding implies that all α_2_δ-1 is proteolytically processed prior to intracellular trafficking into DRG axons. Indeed, our biochemical and cell fractionation data indicate that proteolytic cleavage occurs mainly during trafficking to the plasma membrane (Kadurin et al., 2012) (data not shown). However, this result opens the possibility that uncleaved α_2_δ-1 may reach the cell surface in DRGs cell bodies and have a functional role there.

We then expressed WT α_2_δ-1 in cultured DRG neurons in order to mimic its upregulation in the neuropathic state, and detected a significant amount of pro-α_2_δ-1 in WCL (Figure 6b, lane 2). As expected, transfection of α_2_(3C)δ-1 into DRG neurons resulted in abundant expression of the pro-α_2_(3C)δ-1 form in WCL (Figure 6b, lane 3).

### Uncleaved pro-α_2_δ-1 plays an inhibitory role in the calcium channel function

We then investigated whether uncleaved α_2_δ-1 was inactive, or whether its presence might play an inhibitory role, as suggested by the fact that α_2_(3C)δ-1 increases cell surface expression of Ca_V_2.2 by about 2.5-fold in cell lines, but does not support any increase of Ca_V_2.2 currents. We therefore examined the effect of α_2_(3C)δ-1 expressed in cultured DRG neurons on native calcium channel currents (Figure 6c). We have previously shown that expression of WT α_2_δ-1 in cultured DRG neurons caused an increase of native calcium currents (D'Arco et al., 2015) (shown with additional data in Figure 6d). Here, we found that expression of α_2_(3C)δ-1 produced a marked reduction in endogenous calcium channel currents in DRG neurons (by 43.2% at +10 mV; Figure 6c,d), supporting the hypothesis that pro-α_2_δ-1 inhibits endogenous DRG calcium currents.

Furthermore, when Ca_V_2.2-HA was transfected with β1b and WT α_2_δ-1 into DRG neurons, there was a clear increase in Ca_V_2.2-HA on the cell surface of the DRG cell bodies after 2 days, compared to Ca_V_2.2-HA/β1b alone (Figure 6e,f). In contrast, for co-expression with α_2_(3C)δ-1, the amount of Ca_V_2.2-HA on the plasma membrane was the same as without exogenous α_2_δ, rather than showing any inhibition (Figure 6e,f). This suggests that the inhibitory effect of α_2_(3C)δ-1 on DRG calcium currents (Figure 6c,d) is not due to inhibition of channel trafficking to the somatic plasma membrane.

### Proteolytic cleavage of α_2_δ-1 is required for trafficking of Ca_V_2.2 Into neuronal processes

**Figure 7.**
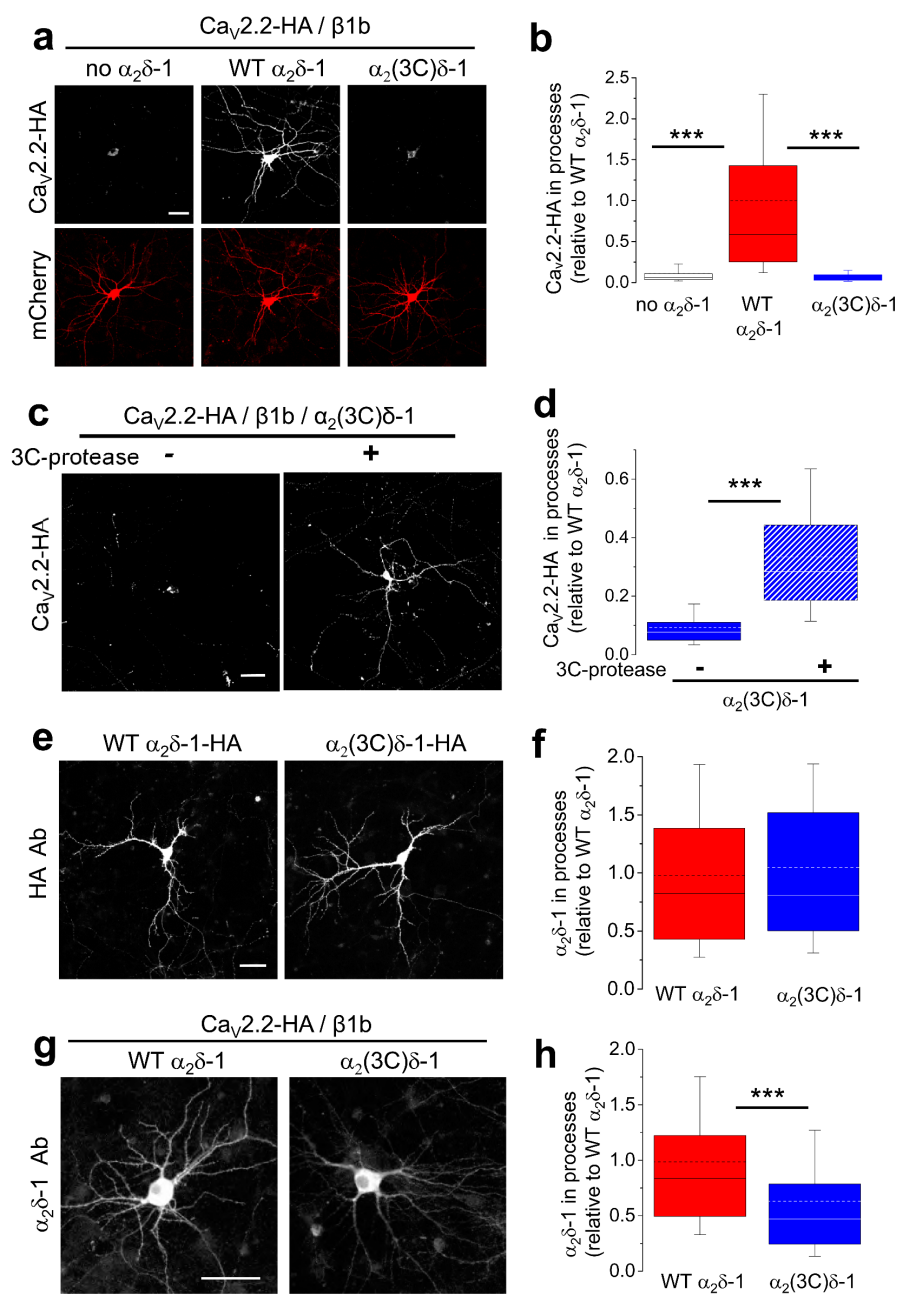
Effect of α_2_δ-1 and proteolytic cleavage of α_2_(3C)δ-1 on trafficking of Ca_V_2.2 into hippocampal neurites. (**a**) Images showing Ca_V_2.2-HA in permeabilized hippocampal neurons (top, white), when co-transfected with β1b, mCherry (bottom, red) and either no α_2_δ (left), WT α_2_δ-1 (middle) or α_2_(3C)δ-1 (right). Scale bar: 50 µm applies to all images. (**b**) Box and whisker plots for Ca_V_2.2-HA expression in processes without α_2_δ (open bar, n = 136 processes from 29 cells), with α_2_δ-1 (red bar, n = 147 processes from 27 cells) or with α_2_(3C)δ-1 (blue bar, n = 109 processes from 22 cells). ***p<0.001, 1 way ANOVA and Bonferroni *post hoc* test. (**c**) Images showing Ca_V_2.2-HA in permeabilized hippocampal neurons (white), when co-transfected with β1b, α_2_(3C)δ-1 and mCherry (transfection marker, not shown), either without (left) or with (right) 3C-protease. Scale bar: 50 µm applies to both images. (**d**) Box and whisker plots for Ca_V_2.2-HA expression in processes with α_2_(3C)δ-1, transfected without (solid blue bar, n = 191 processes), or with 3C-protease (blue hatched bar, n = 187 processes). ***p<0.001, 1 way ANOVA and Bonferroni *post hoc* test. (**e**) Images showing WT α_2_δ-1-HA (left) or α_2_(3C)δ-1-HA (right) expressed in permeabilized hippocampal neurons (white), co-transfected only with mCherry (transfection marker, not shown). Antigen retrieval was used prior to the HA Ab. Scale bar: 50 µm applies to both images. (**f**) Box and whisker plots for expression in the processes of WT α_2_δ-1-HA (red bar, n = 248 processes from 52 cells) and α_2_(3C)δ-1 (blue bar, n = 263 processes from 51 cells). (**g**) Images showing α_2_δ-1 in hippocampal neurons (white), when transfected with Ca_V_2.2-HA, β1b, mCherry (transfection marker, not shown) and either WT α_2_δ-1 (left) or α_2_(3C)δ-1 (right). Antigen retrieval was used prior to the α_2_δ-1 mAb. Scale bar: 50 µm applies to both images. (**h**) Box and whisker plots for α_2_δ-1 expression in hippocampal processes, for WT α_2_δ-1 (red bar, n = 221 processes) and α_2_(3C)δ-1 (blue bar, n = 184 processes). ***p<0.0001, Student’s t test. **DOI:** http://dx.doi.org/10.7554/eLife.21143.020

In view of our finding that all endogenous α_2_δ-1 is proteolytically cleaved in axons but not cell bodies (Figure 6a), we then asked whether proteolytic processing was required for trafficking either of α_2_δ-1 itself, or of associated Ca_V_2.2, into neuronal processes. For this purpose, we used cultured hippocampal neurons which can be transfected after the establishment of extensive neurite outgrowth in culture. We found the surprising result that, when we co-transfected Ca_V_2.2/β1b with either WT α_2_δ-1, α_2_(3C)δ-1 or the corresponding empty vector, WT α_2_δ-1 was essential for trafficking of Ca_V_2.2 into the processes of hippocampal neurons, whereas in the presence of α_2_(3C)δ-1, or without α_2_δ, there was virtually no trafficking of Ca_V_2.2 out of the soma (Figure 7a,b). Strikingly, the inability of α_2_(3C)δ-1 to drive Ca_V_2.2 into neuronal processes was reversed by co-expression of 3C-protease (Figure 7c,d).

**Figure 7—figure supplement 1.**
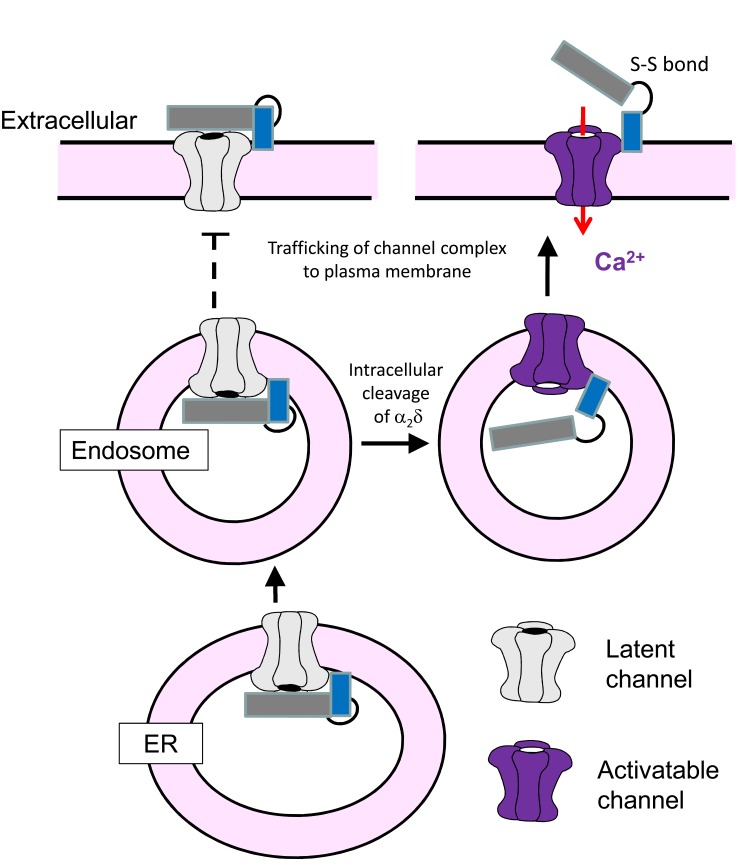
Cartoon showing processing and trafficking of α_2_δ in neurons. Cartoon of the effect of proteolytic processing of α_2_δ (dark gray α_2,_ blue δ) on Ca_V_2.2 trafficking and voltage-dependent activation. The gray channel is one that cannot be activated by depolarization, whereas the purple channel is functional. The solid arrows indicate the likely trafficking pathway in neurons. The dotted lines show that trafficking of channels containing uncleaved α_2_δ to the plasma membrane and into processes, and extracellular proteolytic cleavage of α_2_δ indicates do not occur in native neurons studied here, although it is possible that this may occur in pathological conditions, for example following α_2_δ-1 upregulation as a result of neuropathic nerve injury in DRG neurons. **DOI:** http://dx.doi.org/10.7554/eLife.21143.021

The failure of Ca_V_2.2 to traffic into the neurites in the presence of α_2_(3C)δ-1 was not primarily due to a defect in the trafficking of the uncleaved α_2_δ subunit itself, since, when the α_2_δ subunits were transfected alone, WT α_2_δ-1 and α_2_(3C)δ-1 were both able to extensively access the hippocampal neurite compartment (Figure 7e,f). In contrast, when the localization of the α_2_δ subunits was examined following co-transfection with Ca_V_2.2/β1b, the trafficking of α_2_(3C)δ-1 into neurites was 37% lower than that of WT α_2_δ-1 (Figure 7g,h), suggesting it has been retained by interaction with Ca_V_2.2. Similar results to those with α_2_δ-1 were obtained when comparing the ability of WT α_2_δ-3 and α_2_(3C)δ-3 to traffic Ca_V_2.2 into neurites, which was also reversed by 3C-protease (data not shown). This further confirmed that the association of Ca_V_2.2 with mature processed α_2_δ subunits is essential for trafficking of the complex into hippocampal neurites (Figure 7—figure supplement 1).

### Proteolytic cleavage of α_2_(3C)δ-1 enhances Ca^2+^ entry into hippocampal boutons

**Figure 8.**
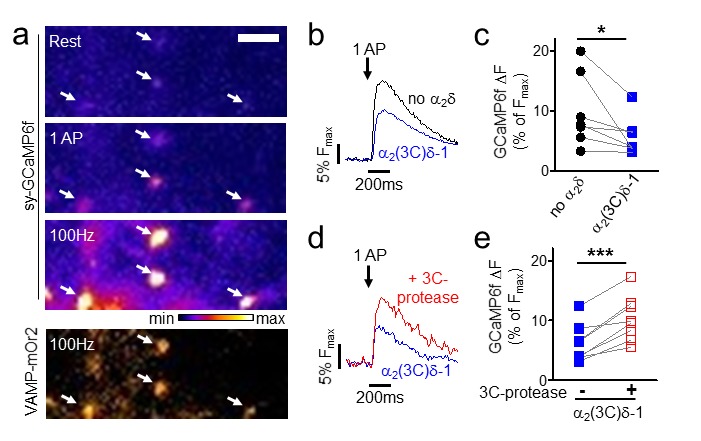
Effect of proteolytic cleavage of α_2_(3C)δ-1 on Ca^2+^ influx in presynaptic terminals of hippocampal neurons. (**a**) Fluorescence changes in presynaptic terminals of hippocampal neurons expressing sy-GCaMP6f and VAMP-mOr2 in response to electrical stimulation. White arrows point to transfected boutons. Top three panels show sy-GCaMP6f fluorescence: at rest (top), after 1 AP (middle) and after 100 Hz stimulation for 1 s (bottom). The bottom panel shows VAMP-mOr2 fluorescence after 100 Hz stimulation for 1 s. Scale bar 5 µm. The pseudocolour scale is shown below the third panel. (**b**) Mean example traces from the same experiment of sy-GCaMP6f fluorescence changes in response to 5 single APs from individual presynaptic terminals of neurons co-transfected with either empty vector (black trace) or α_2_(3C)δ-1 (blue trace). (**c**) Sy-GCaMP6f fluorescence changes (expressed as % of F_max_) in response to 1 AP from boutons co-transfected with either empty vector (black filled circles) or α_2_(3C)δ-1 (blue filled squares) (n = 7 independent experiments, *p=0.049, paired t test). (**d**) Mean example traces of sy-GCaMP6f fluorescence changes in response to 5 single APs from presynaptic terminal of neurons co-transfected with either α_2_(3C)δ-1 (blue trace) or α_2_(3C)δ−1 + 3C-protease (red trace). (**e**) Sy-GCaMP6f fluorescence changes (expressed as % of F_max_) in response to 1 AP from boutons co-transfected with either α_2_(3C)δ-1 (blue filled squares) or α_2_(3C)δ−1 + 3C-protease (red open squares) (n = 8 independent experiments, ***p=0.0005, paired t test). **DOI:** http://dx.doi.org/10.7554/eLife.21143.022

We then examined the effect of expression of α_2_(3C)δ-1 alone in hippocampal neurons on action potential (AP)-induced Ca^2+^ entry into presynaptic boutons. These were identified by co-expressed vesicle-associated membrane protein-mOrange2 (VAMP-mOr2), and Ca^2+^ transients were measured using synaptophysin-GCaMP6f (sy-GCaMP6f, Figure 8a). The uncleaved α_2_(3C)δ-1 reduced Ca^2+^ entry in response to single AP stimulation in all experiments performed (Figure 8b,c), to 65.8 ± 9.1% (n = 7, p=0.0093, 1 sample t test) of control. The inhibitory effect of α_2_(3C)δ-1 on the response to a single AP was reversed by co-expression of 3C-protease in all experiments performed (Figure 8d,e), an increase of 81.5 ± 16.1% (n = 8, p<0.0001, 1 sample t test).

## Discussion

In this study, we have identified key roles for proteolytic processing of pro-α_2_δ subunits. Our main findings are: firstly that proteolytic cleavage of pro-α_2_δ represents an essential step for the expression of mature functional calcium channels. Here we show, using a combination of techniques, that the appearance of functional Ca_V_2.2 channels associated with the proteolytic processing of α_2_δ subunits, occurs independently of changes in channel trafficking to the plasma membrane in undifferentiated cell lines. A key experiment is the demonstration that acute restoration of voltage-dependent activation by proteolytic cleavage of pro-α_2_δ can be induced at the cell surface by extracellularly applied protease. Secondly, we find that lack of proteolytic cleavage of α_2_δ-1 represents a very significant barrier to trafficking of Ca_V_2.2 channels into cultured hippocampal neuronal processes. Trafficking of the channel complex into neurites is dependent on an effect of mature cleaved α_2_δ on Ca_V_2.2, rather than due primarily to the differential ability of mature α_2_δ to be trafficked and uncleaved pro-α_2_δ to be retained in the soma. This is clearly shown by the fact that uncleaved α_2_(3C)δ-1 is fully able to traffic alone into neurites. Thirdly, we provide evidence for an inhibitory role for the pro-form of α_2_δ-1. It is highly likely that proteolytic cleavage of α_2_δ could induce a conformational change, which would impact on its interaction with the α_1_subunit. The recent structure of the Ca_V_1.1 complex resolves the domain structure of the α_2_δ-1 subunit to contain the VWA domain and four tandem cache domains (Wu et al., 2016). The δ subunit contributes part of the fourth cache domain, and it is therefore possible that the domain organization would be modified by proteolytic cleavage of α_2_δ.

In a previous study, we showed that WT α_2_δ-1 increases the amount of Ca_V_2.2 on the cell surface in N2A cells, by about two-fold (Cassidy et al., 2014). We have also found that heterologously-expressed α_2_δ subunits are only partially processed into α_2_ and δ in all expression systems examined, although proteolytic cleavage was much more marked at the plasma membrane (Davies et al., 2010; Kadurin et al., 2012). The incomplete cleavage of heterologously expressed WT α_2_δ is in contrast to the complete processing of muscle and brain α_2_δ-1 (Jay et al., 1991; Patel et al., 2013) and cerebellar α_2_δ-2 (Davies et al., 2006), and likely represents saturation of the processing enzyme(s). Thus, it was clear that determining the role of proteolytic cleavage of α_2_δ on calcium channel function would require additional strategies. A previous study found that various mutations around the cleavage site reduced, but did not abolish, calcium current enhancement by α_2_δ-1 subunits (Andrade et al., 2007), leaving the role of proteolytic cleavage of α_2_δ-1 an open question. Here we show conclusively that for both α_2_δ-1 and α_2_δ-3, mutations that prevent their cleavage into α_2_ and δ do not prevent the appearance of pro-α_2_δ on the cell surface. Furthermore, in the cell lines examined here, but not in neurons, Ca_V_2.2 trafficking to the plasma membrane was enhanced by an uncleaved pro-form of α_2_δ-1 to a similar extent as for WT α_2_δ-1, utilising a mechanism that is independent of the plasma membrane potential. However, this led to the increased cell surface expression of a calcium channel complex which appeared to be non-functional, since the uncleaved pro-α_2_δ did not enhance Ca_V_2.2 calcium currents. Thus trafficking of Ca_V_2.2 to the plasma membrane by α_2_δ-1 subunits in cell lines can be uncoupled from the functional effects of α_2_δ subunits on voltage-dependent activation of the channels.

Previous in vitro studies have examined the effect of α_2_δ subunits on calcium channel currents resulting from several combinations of Ca_V_α1 and β subunits. In whole-cell current recordings, α_2_δ subunits increase the maximum conductance from 3 to 10-fold, depending on subunit combination and conditions used (Canti et al., 2005; Mori et al., 1991; Klugbauer et al., 1999; Hobom et al., 2000; Gao et al., 2000; Barclay et al., 2001; Hendrich et al., 2008). However, they have no effect on single channel conductance, and for Ca_V_2 channels there are only minor effects of α_2_δ subunits on most parameters relating to open probability (Hoppa et al., 2012; Barclay et al., 2001; Brodbeck et al., 2002; Wakamori et al., 1999; Meir and Dolphin, 1998), which would be unlikely to account for the large increase in whole-cell conductance. By contrast, a finding that is consistent with the increase in whole-cell current, is that the fraction of null traces was markedly reduced by α_2_δ-1 in unitary current recordings from oocytes expressing Ca_V_2.2/β1b/α_2_δ-1 (9% null traces), a 3–4-fold decrease, compared with those expressing Ca_V_2.2 alone (39% null traces) or Ca_V_2.2/β1b (28% null traces) (Wakamori et al., 1999). This observation suggests that α_2_δ shifts the equilibrium towards active modes of the channel, from an inactive null mode represented by the long closed state. Furthermore, it has recently been shown that α_2_δ-1 promotes voltage sensor movement of Ca_V_1.2 (Savalli et al., 2016), thus hyperpolarizing channel activation (Savalli et al., 2016). Although the interaction of α_2_δ subunits with Ca_V_2 channels may differ, as there is not such a clear shift in the voltage-dependence of activation (Kadurin et al., 2012; Canti et al., 2005), it is tempting to speculate that the association of the channels with uncleaved pro-α_2_δ might interfere with voltage sensor movement.

We have found previously that the MIDAS motif in the VWA domains of α_2_δ-1 and α_2_δ-2 subunits is key to increasing calcium channel function (Hoppa et al., 2012; Cassidy et al., 2014; Canti et al., 2005). The recent structure of Ca_V_1.1 indicates that the VWA domain MIDAS motif of α_2_δ-1 closely associates with the loop between S1 and S2 in Domain I of the α1S subunit (Wu et al., 2015). The VWA domain is present in all α_2_δ subunits, but it only has a perfect MIDAS motif in α_2_δ-1 and α_2_δ-2 (Dolphin, 2013). Thus α_2_δ-3 has an incomplete MIDAS motif, which is predicted to have reduced function (Whittaker and Hynes, 2002), and indeed α_2_δ-3 has less effect on cell surface expression of Ca_V_2.2 than α_2_δ-1 in the present study (compare Figures 2c and 4b).

In agreement with the hypothesis that pro-α_2_δ is an inhibitory gate-keeper for calcium channel function, we observed here that an uncleaved α_2_δ-1 construct (α_2_(3C)δ-1) had an inhibitory effect on endogenous DRG neuron calcium currents, and on presynaptic Ca^2+^ entry in hippocampal neuronal boutons, which could be reversed by co-expression of the 3C-protease. This is in contrast to a previous study in which overexpression of WT α_2_δ subunits, which will form a mixture of both cleaved and uncleaved α_2_δ (Figure 6b), reduced Ca^2+^ entry at synapses (Hoppa et al., 2012). Our result indicates that proteolytic processing of α_2_δ-1 represents an essential functional checkpoint to allow channel activation by depolarization, and the pro-α_2_δ species inhibits function.

Furthermore, we show here that native pro-α_2_δ-1 can be observed in the cell bodies of DRG neurons, but in the axons it is completely processed to α_2_ and δ; this is despite the fact that it is still present in intracellular trafficking vesicles (Bauer et al., 2009). It is of interest in this regard that although α_2_δ-1 is elevated in all DRG neurons following nerve injury (Bauer et al., 2009; Luo et al., 2001), calcium currents from DRGs extracted after nerve injury show a variable decrease in calcium currents (Hogan et al., 2000; McCallum et al., 2006, 2011). This would agree with the present finding that somatic DRG α_2_δ-1 upregulated after nerve injury remains in part uncleaved, and that only when in its mature processed form can it function to increase calcium currents and trafficking of calcium channels into neurites.

The functional importance of proteolytic cleavage of α_2_δ subunits is further emphasised by our finding that the trafficking of Ca_V_2.2 into the processes of cultured hippocampal neurons is completely prevented by the uncleaved α_2_(3C)δ-1, and this is reversed by its intracellular proteolytic cleavage with the 3C protease. Thus, the proteolytic processing of α_2_δ represents an essential checkpoint for neuronal trafficking of calcium channels, to ensure that only mature channel complexes capable of voltage-dependent activation reach specific plasma membrane domains such as presynaptic terminals (Figure 7—figure supplement 1). We have shown previously that the small GTPase, rab11, is involved in α_2_δ-mediated trafficking of calcium channels (Tran-Van-Minh and Dolphin, 2010), and rab11, among many other proteins, is important for vesicular cargo transport into neurites (Villarroel-Campos et al., 2016). We have also identified the importance of adaptor protein-1 in Ca_V_2.2 trafficking (Macabuag and Dolphin, 2015). It will be of great interest in future studies to determine the additional mechanisms present in neurons, in contrast to non-neuronal cell lines, restricting the cargo transport into neurites to activatable calcium channel complexes in which α_2_δ subunits are proteolytically cleaved.

## Materials and methods

### Molecular biology

The cDNAs used were: rat α_2_δ-1 (M86621) and mouse α_2_δ-3 (AJ010949), rabbit Ca_V_2.2 (D14157 without 3' UTR), and rat β1b (Tomlinson et al., 1993). In some experiments Ca_V_2.2 was used with an N-terminal GFP (Macabuag and Dolphin, 2015), or containing an extracellular BBS tag or HA tag (Cassidy et al., 2014). α_2_δ-1-HA (HA-tag sequence YPYDVPDYA inserted between Asn-549 and Asp-550) (Kadurin et al., 2012) and α_2_δ-3-HA (HA between Lys-595 and Arg-596) were used in all imaging and other experiments except when the subunits were co-expressed with Ca_V_2.2-HA in hippocampal neurons, in which case untagged α_2_δ-1 or α_2_δ-3 were used. Both α_2_δ-HA constructs showed normal function in terms of enhancing Ca_V_ currents ([Kadurin et al., 2012] and data not shown). Proteolytic cleavage site mutations were made as indicated (1b and 4c). GFP-β1b was used in some electrophysiological experiments (Waithe et al., 2011). Human TASK3 (*KCNK9*) cDNA (NM_001282534) was obtained from Prof. A Mathie. The cDNAs were in the pMT2 vector for expression in tsA-201 cells, in pcDNA3 for expression in N2A cells and pcDNA3 or pCAGGS for expression in DRG or hippocampal neurons, respectively. In our hands expression of the large constructs encoding Ca_V_2.2 and α_2_δ in pcDNA3 was very poor in hippocampal neurons (data not shown), whereas expression from the pCAGGS vector, containing chicken β-actin promoter, was strong and sustained. The cDNAs encoding Human Rhinovirus (HRV)−3C protease or mutated 3C (in which the active site Cys-147 was mutated to Val) were first subcloned into the pHLSec vector (Yurtsever et al., 2012; Aricescu et al., 2006), using Age I and Kpn I sites in frame with an N-terminal signal sequence, to include the signal sequence from pHLSec, and then into pMT2 and pCAGGS vectors for expression in cell lines or hippocampal neurons. VAMP-mOr2 was generated by replacing mCherry from pCAGGs-VAMPmCherry (gift from Dr. TA Ryan) with mOrange2. Sy-GCaMP6f was made by replacing GCaMP3 in pCMV-SyGCaMP3 (gift from Dr. TA Ryan) by GCaMP6f (Chen et al., 2013). The cDNA for mut2-GFP (Cormack et al., 1996) in pMT2 was used as a negative control in co-immunoprecipitation (co-IP) experiments. Site-directed mutagenesis was carried out using standard procedures, and all subcloning and mutations confirmed by sequencing.

### Antibodies and other materials

Ca channel antibodies (Abs) used were: α_2_δ-1 Ab (mouse monoclonal against α_2_-1 moeity, Sigma-Aldrich, epitope identified in [Cassidy et al., 2014]), α_2_-3 (71–90) Ab (rabbit; polyclonal) (Davies et al., 2010), δ-3 (1035–1049) Ab (Davies et al., 2010), anti-Ca_V_2.2 II-III loop Ab (rabbit polyclonal) (Raghib et al., 2001). Other Abs used were anti-HA (rat monoclonal, Roche), anti-HA (rabbit polyclonal, Sigma), anti-GAPDH Ab (mouse monoclonal, Ambion), anti-flotillin-1 (mouse monoclonal, BD Biosciences), and GFP Ab (Living Colors, rabbit polyclonal; BD Biosciences). For immunocytochemistry, secondary Abs (1:500) used were anti-rabbit-Alexa Fluor 594, anti-rat-Alexa Fluor 594, anti-mouse-Alexa Fluor 647 (Life Technologies) or fluorescein isothiocyanate (FITC)-anti-rat (Sigma-Aldrich). The following secondary Abs were used for western blot: goat anti-rabbit, goat anti-rat and goat anti-mouse Abs coupled to horseradish peroxidase (HRP) (Biorad, Hemel Hempstead, UK). The signal was obtained by HRP reaction with fluorescent product (ECL 2; Thermo Scientific) and membranes were scanned on a Typhoon 9410 phosphorimager (GE Healthcare). Lyophilized active thrombin was obtained from Sigma, suspended in filter-sterilised PBS (Sigma; pH7.4) to 1000 U/ml and frozen in aliquots until use.

### Cell culture, transfection and enzymatic treatment

Cell lines were plated onto cell culture flasks, coverslips or glass-bottomed dishes (MatTek Corporation, Ashland, MA), coated with poly-L-lysine, and cultured in a 5% CO_2_ incubator at 37°C. The human embryonic kidney tsA-201 cells (European Collection of Authenticated Cell Cultures, # 96121229), tested to be mycoplasma-free, were cultured in Dulbecco’s modified Eagle’s medium (DMEM) supplemented with 10% foetal bovine serum (FBS), 1 unit/ml penicillin, 1 μg/ml streptomycin and 1% GlutaMAX (Life Technologies, Waltham, MA). tsA-201 cells were transfected using Fugene6 (Promega, Fitchburg, WI), according to the manufacturer’s protocol. The enzymatic treatments with 30 U/ml Thrombin protease (Sigma) diluted in DMEM without supplements were done for indicated times in a 5% CO_2_ incubator at 37°C. Mouse neuroblastoma N2A cells (American Tissue culture collection, # CCL-131) were obtained from Professor Roger Morris, Kings College London (Parkyn et al., 2008), and were tested to be mycoplasma-free. They were cultured in 50% DMEM and 50% OPTI-MEM supplemented with 5% FBS, 1 unit/ml penicillin, 1 μg/ml streptomycin, and 1% GlutaMAX. N2A cells were transfected using PolyJet (SignaGen Laboratories, Gaithersburg, MD), according to the manufacturer’s protocol.

### Neuronal culture and transfection

DRG neurons were isolated from P10 male Sprague Dawley rats and transfected essentially as described recently (D'Arco et al., 2015) with an Amaxa Nucleofector (Lonza, Basel, Switzerland) according to the manufacturer’s protocol. Transfected neurons were plated onto coverslips coated with poly-L-lysine, and cultured in DMEM/F12 supplemented with 10% FBS, 1 unit/ml penicillin, 1 μg/ml streptomycin, 1% GlutaMAX and 100 ng/ml NGF in a 5% CO_2_ incubator at 37°C. Hippocampal neurons were obtained from P0 rat pups as previously described (Morales et al., 2000). Approximately 75 × 10^3^ cells in 100 μl of plating solution (Neurobasal medium supplemented with B27 (Life Technologies; 2%), HEPES (10 mM), horse serum (5%), glutamine (0.5 mM), and 1 unit/ml penicillin, 1 μg/ml streptomycin) were seeded onto sterile poly-lysine-coated glass coverslips. After 2 hr, the plating solution was replaced with 1 ml of growth medium (serum-free Neurobasal medium supplemented with B27 (Life Technologies; 4%), 2-mercaptoethanol (25 μM), glutamine (0.5 mM), and 1 unit/ml penicillin, 1 μg/ml streptomycin), half of which was replaced every 3–4 days. At 7 days in vitro (DIV) and 2 hr before transfection, half of the medium was removed, and kept as ‘conditioned’ medium and 500 μl of fresh medium was added. The hippocampal cell cultures were then transfected with Lipofectamine 2000 according to the manufacturer’s protocol using 2 μg of cDNA mix per well (Invitrogen). After 2 hr, the transfection mixes were replaced with growth medium consisting of 50% ‘conditioned’ and 50% fresh medium. The cultures were used for immunostaining experiments at 14 DIV, or for live imaging as described below.

### Cell surface biotinylation, Cell lysis, deglycosylation and immunoblotting

The procedures were modified from those described in more detail previously (Davies et al., 2010; Page et al., 2004). Briefly, 72 hr after transfection, tsA-201 cells were incubated for 30 min at room temperature with 0.5 mg/ml Premium Grade EZ-link Sulfo-NHS-LC-Biotin (Thermo Scientific) in PBS and the reaction was quenched with 200 mM glycine. The cells were incubated for 45 min on ice in PBS, pH 7.4 at 4°C containing 1% Igepal; 0.1% SDS and protease inhibitors (PI, cOmplete, Roche; used according to manufacturer’s instructions), to allow cell lysis. The WCL was then centrifuged at 18,000 ×g for 20 min at 4°C and the pellet discarded. The supernatant was assayed for total protein (Bradford assay, Biorad). Immunoblot analysis was performed essentially as described previously (Kadurin et al., 2012). Cleared WCL corresponding to 20–40 µg total protein was diluted with Laemmli sample buffer (Davies et al., 2010) supplemented with 100 mM dithiothreitol (DTT), incubated at 60°C for 10 min and resolved by SDS-polyacrylamide gel electrophoresis (PAGE) on 3%–8% Tris-Acetate or 4–12% Bis-Tris gels (Invitrogen) and transferred to polyvinylidene fluoride (PVDF) membrane (Biorad) by western blotting. When required the membrane was cut and incubated with different antibodies. Biotinylated lysates (adjusted to between 0.5 and 1 mg/ml total protein concentration) were applied to 40 µl prewashed streptavidin-agarose beads (Thermo Scientific) and rotated overnight at 4°C. The beads were then washed 3 times with PBS containing 0.1% Igepal and, when required, the streptavidin beads were re-suspended in PNGase-F buffer (PBS, pH 7.4, supplemented with 75 mM β-mercaptoethanol, 1% Igepal, 0.1% SDS, and PI) and deglycosylated for 3 hr at 37°C with 1 unit of PNGase-F (Roche Applied Science) added per 10 μl volume. The samples were then resuspended in an equal volume of 2 × Laemmli buffer with 100 mM DTT, followed by 10 min incubation at 60°C. The eluted protein was analysed by immunoblotting, as described above. GAPDH is ~39 kDa and does not resolve well in 3–8% Tris-Acetate gels. Therefore, in cases when 3–8% gels were used to resolve high MW proteins, which required GAPDH as negative control for biotinylated fractions, the same samples were re-loaded on a 4–12% Bis-Tris gel to analyse separately.

### Co–immunoprecipitation

The protocol was adapted from a procedure described previously (Gurnett et al., 1997). Briefly, a tsA-201 cell pellet derived from one confluent 75-cm^2^ flask was resuspended in a co-IP buffer (20 mM HEPES (pH 7.4), 300 mM NaCl, 1% Digitonin and PI), sonicated for 8 s at 20 kHz and rotated for 1 hr at 4°C. The samples were then diluted with an equal volume of 20 mM HEPES (pH 7.4), 300 mM NaCl with PI (to 0.5% final concentration of Digitonin), mixed by pipetting and centrifuged at 18,000 ×g for 20 min. The supernatants were collected and assayed for total protein (Bradford assay; Biorad). 1 mg of total protein was adjusted to 2 mg/ml with co-IP buffer and incubated overnight at 4°C with anti-GFP polyclonal antibody (1:200; BD Biosciences). 30 μl A/G PLUS Agarose slurry (Santa Cruz) was added to each tube and further rotated for 2 hr at 4°C. The beads were then pelleted by 500 ×g centrifugation at 4°C and washed three times with co-IP buffer containing 0.2% Digitonin. The beads were then resuspended in an equal volume of PNGase-F buffer and proteins were deglycosylated with PNGase-F as described above. Aliquots of the initial WCL prior to co-IP were also deglycosylated in parallel; 2 × Laemmli buffer with 100 mM DTT was added and samples were analysed by SDS-PAGE and western blotting.

### Preparation of triton X-100-insoluble membrane fractions (DRMs)

The protocol was similar to that described previously (Davies et al., 2010; Kadurin et al., 2012). All steps were performed on ice. Confluent tsA-201 cells from two 175-cm (Kurshan et al., 2009) flasks were taken up in Mes-buffered saline (MBS, 25 mm Mes, pH 6.5, 150 mm NaCl, and PI) containing 1% (v/v) Triton X-100 (TX-100) (Thermo Scientific), and left on ice for 1 hr. An equal volume of 90% (w/v) sucrose in MBS was then added to a final concentration of 45% sucrose. The sample was transferred to a 13 ml ultracentrifuge tube and overlaid with 10 ml of discontinuous sucrose gradient, consisting of 35% (w/v) sucrose in MBS (5 ml) and 5% (w/v) sucrose in MBS (5 ml). The sucrose gradients were ultra-centrifuged at 140,000 ×*g*_avg_ (Beckman SW40 rotor) for 18 hr at 4°C. 1 ml fractions were subsequently harvested from the top to the bottom of the tube and aliquots of 10 μl from each fraction were analysed by SDS-PAGE and western blotting to obtain DRM profiles. When necessary, DRMs (combined peak fractions identified by the presence of flotillin-1) from the gradient were washed free of sucrose by dilution into 25 volumes of cold PBS (pH 7.4) and pelleted by ultracentrifugation at 150,000 ×*g* (Beckman Ti 70 rotor) for 1 hr at 4°C. TX-100-insoluble protein was resuspended in appropriate buffers as described for ^3^H-gabapentin binding or for deglycosylation as described above.

### Immunocytochemistry, imaging and analysis

The procedure in tsA-201 and N2A cells was performed essentially as described previously with minor modifications (Davies et al., 2010; Kadurin et al., 2012). Briefly, 48 hr post-transfection the cells were fixed with 4% paraformaldehyde (PFA) in PBS, pH7.4 at 20°C for 5 min, and then incubated for PBS for 15 min, which contained 0.1% TX-100 if permeabilization was applied. Blocking was performed for 1 hr at 20°C in PBS containing 20% goat serum and 5% bovine serum albumen (BSA). The indicated primary antibodies were then applied (diluted in PBS with10% goat serum and 2.5% BSA) overnight at 4°C or for 1 hr at 20°C. In live-labelling experiments, cells were washed with Krebs Ringer HEPES (KRH) buffer, labelled with α-bungarotoxin (BTX)-AF 488 (Invitrogen; 1:100 in KRH buffer) at 17°C for 30 min, then washed with KRH and fixed as described above. The indicated secondary antibodies were applied (1:500 dilution in PBS, containing 2.5% BSA and 10% goat serum) at 20°C for 1 hr. Cell nuclei were stained with 0.5 µM 4’,6’-diamidino-2-phenylindole (DAPI) in PBS for 5 min. The coverslips were mounted onto glass slides using VECTASHIELD mounting medium (Vector Laboratories, Peterborough, UK). Cultures of transfected hippocampal neurons were fixed after 14 DIV in PBS containing 4% PFA/4% sucrose for 5 min at 20°C, and then the procedure was as described above. In some cases, where stated, an antigen retrieval step was performed between the fixation and blocking steps: the cells were incubated for 10 min at 95°C in 10 mM citrate buffer (pH 6) containing 0.05% Tween 20.

Imaging was performed on Zeiss LSM 780 confocal microscope as described in more detail elsewhere (Davies et al., 2010; Kadurin et al., 2012). Images were obtained at fixed microscope settings for all experimental conditions of each experiment. Images of N2A and tsA-201 cells were obtained using a 63 × objective at a resolution of 1024 × 1024 pixels and an optical section of 0.8–1 μm. After choosing a region of interest containing transfected cells, the 3 × 3 tile function of the microscope allowed imaging of a larger area selected without bias. Every cell identified as transfected was included in the measurements, to ensure lack of bias.

Images of tsA-201 and N2A cells were analyzed using imageJ (*imagej.net*) using a modification of the procedure described previously (Davies et al., 2010; Kadurin et al., 2012). Surface labelling in non-permeabilized or total staining in permeabilized cell bodies was measured using the freehand line tool and manually tracing the surface of the cell or drawing around the cell (omitting the nucleus) respectively. The value of the mean pixel intensity in different channels was measured separately and background was subtracted by measuring the intensity of an imaged area without transfected cells. All data were then normalized to the appropriate positive control for each experiment before combining experiments.

Hippocampal neurons were imaged using a 20 × objective with a 5 μm optical section. Large tiles were manually selected following all processes expressing mCherry. The fluorescence intensity along neuronal projections of hippocampal neurons was assessed in FIJI as follows: a circle of 100 μm diameter was drawn around each neuronal cell body. A free-hand line (2 μm thick; ~ 30 μm long) was drawn along the neurite extending beyond the circle and the mean grey intensity of all the pixels within the line was measured in both channels corresponding to the fluorescence of HA or α_2_δ-1 immunostaining and mCherry.

### Analysis of α_2_δ-1 in dorsal root ganglion (DRG) neurons and axons

Immunoblotting of DRG tissue and associated nerves was performed as described previously (Bauer et al., 2009). Tissue was taken from rats 4d following a spinal nerve ligation procedure, performed in the course of a previous study (Bauer et al., 2009), in order to increase the amount of α_2_δ-1 protein in the harvested tissue. Tissue from DRGs, sections of spinal nerve and dorsal roots were pooled from 4 rats, and stored at −80°C until use in this study. For deglycosylation, protein samples were diluted in PBS + 1% Igepal + 1% β-mercaptoethanol and treated with 1 unit of PNGase F overnight at 37°C.

### ^3^H gabapentin binding assay

Binding of ^3^H-gabapentin to DRM preparations was carried out, essentially as previously described (Lana et al., 2014), in a final volume of 250 µl at room temperature for 45 min. DRM fractions (4 µg of protein per tube) were incubated with various concentrations of [^3^H]-gabapentin (specific activity 36 Ci/mmol, American Radiolabeled Chemicals, St. Louis, MO, USA) in 10 mM HEPES/KOH pH 7.4, then rapidly filtered through GF/B filters, pre-soaked with 0.3% polyethyleneimine. Filters were washed three times with 3 ml ice-cold 50 mM Tris/HCl, pH 7.4 and counted on a scintillation counter. Concentrations of [^3^H]-gabapentin greater than 50 nM were achieved by adding non-radioactive gabapentin and correcting the specific binding by the dilution factor (Canti et al., 2005; Lana et al., 2014). Non-specific binding was determined in the presence of 20 µM non-radioactive gabapentin. Data points were determined in triplicate for each experiment, and data for each experiment were analysed by fitting specific binding to the Hill equation (Lana et al., 2014).

### Electrophysiology

Calcium channel currents in transfected tsA-201 cells were investigated by whole cell patch clamp recording, essentially as described previously (Berrow et al., 1997). The patch pipette solution contained in mM: Cs-aspartate, 140; EGTA, 5; MgCl_2_, 2; CaCl_2_, 0.1; K_2_ATP, 2; Hepes, 10; pH 7.2, 310 mOsm with sucrose. The external solution for recording Ba^2+^ currents contained in mM: tetraethylammonium (TEA) Br, 160; KCl, 3; NaHCO_3_, 1.0; MgCl_2_, 1.0; Hepes, 10; glucose, 4; BaCl_2_, 1,or 2 as indicated, pH 7.4, 320 mosM with sucrose. Unless otherwise stated, 1 mM extracellular Ba^2+^ was the charge carrier. Pipettes of resistance 2–4 MΩ were used. An Axopatch 1D or Axon 200B amplifier was used, and whole cell voltage-clamp recordings were sampled at 10 kHz frequency, filtered at 2 kHz and digitized at 1 kHz. 70–80% series resistance compensation was applied and all recorded currents were leak subtracted using P/8 protocol. For DRG neurons, whole cell voltage clamp experiments were performed in small (<19 pF) and medium (20–38 pF) neurons. Membrane potential was held at –80 mV for experiments in tsA-201 cells and −90 mV for DRG experiments. Cells were accepted where the access resistance was less than 5 MΩ, the inward current was > −3 pA/pF at +10 mV, a complete *IV* relationship was obtained and there was no evidence of poor voltage clamp. Analysis was performed using Pclamp 9 (Molecular Devices) and Origin 7 (Microcal Origin, Northampton, MA). *IV* relationships were fit by a modified Boltzmann equation as follows: I=G_max_*(V−V_rev_)/(1+exp(−(V−V_50, act_)/k)) where *I* is the current density (in pA/pF), *G*_max_ is the maximum conductance (in nS/pF), *V*_rev_ is the apparent reversal potential, *V*_50, act_ is the midpoint voltage for current activation, and *k* is the slope factor. Recordings of resting membrane potential were performed as previously described (Margas et al., 2016).

### Live cell imaging

Hippocampal neurons were transfected with VAMP-mOr2 and sy-GCaMP6f, together with the other cDNAs used at 7 DIV. Neurons were imaged after 14–21 DIV. Coverslips were mounted in a laminar-flow perfusion and stimulation chamber (Warner Instruments) on the stage of an epifluorescence microscope (Axiovert 200 M, Zeiss). White and 470 nm LEDs served as light sources (Cairn Research, UK). Fluorescence excitation and collection was performed through a 40 × 1.3 NA Fluar Zeiss objective using 450/50 nm excitation and 510/50 nm emission and 480 nm dichroic filters, and a 545/25 nm excitation and 605/70 nm emission and 565 nm dichroic filters (for mOrange2). Live cell images were acquired as previously described with minor modifications (Margas et al., 2016; Ferron et al., 2014) with an Andor iXon+ (model DU-897U-CS0-BV) back-illuminated EMCCD camera. Fluorescence was collected at 100 Hz over a 512 × 266 pixel area (7 ms integration time). Cells were perfused (0.5 ml min^−1^) in a saline solution at 22°C containing (in mM) 119 NaCl, 2.5 KCl, 2 CaCl_2_, 2 MgCl_2_, 25 HEPES (buffered to pH 7.4), 30 glucose, 10 µM 6-cyano-7-nitroquinoxaline-2,3-dione (CNQX) and 50 µM D,L-2-amino-5-phosphonovaleric acid (AP5). Neurons were stimulated by passing 1 ms current pulses through the field stimulation chamber via platinum electrodes. Neurons expressing syn-GCaMP6f were identified by stimulating the preparation at 33 Hz for 180 ms every 4 s. Subsequently, single stimulations of 1 ms (mimicking a single AP) were repeated 5 times at 45 s intervals. Functional synaptic boutons were identified by the increase of fluorescence of VAMP-mOr2 in response to a 100 Hz stimulation for 1 s. Changes in GCaMP6f fluorescence were normalized to the maximum fluorescence (F_max_) measured in the presence of 5 µM ionomycin and 2 mM Ca^2+^ in the perfusion solution. Analysis was performed with ImageJ (http://rsb.info.nih.gov/ij), using a custom-written plugin (http://rsb.info.nih.gov/ij/plugins/time-series.html). Regions of interest (ROI) of 2 µm diameter were selected according to their responsiveness to a 100 Hz stimulation for 1 s (on average, 20 to 100 ROIs were analyzed per field of view). Peak fluorescence in response to mean of 5 single AP stimuli was determined by averaging 5–10 points of the plateau phase and subtracting the average of 10 points of the baseline before stimulation.

### Data analysis

Data are given as mean ± SEM, or scatter plots, or box (25–75%) and whisker (10–90%) plots with mean and median (dashed and solid lines). Statistical comparisons were performed using either Student’s t test, paired t test, 1-sample t test, ANOVA with appropriate post-hoc test, or Krushkal-Wallis test with appropriate post-hoc test, as stated, using Graphpad Prism 5. Details of tests are given in Supplementary statistics file.

## Additional files

Supplementary file 1. Summary of statistics shown for the quantitative data in the Figures. The statistical data were obtained using Graphpad Prism 5. **DOI:** http://dx.doi.org/10.7554/eLife.21143.023
