# Supplementary material for: Proteolytic maturation of α2δ represents a checkpoint for activation and neuronal trafficking of latent calcium channels
Source: eLife. 2016 Oct 26;5:e21143. doi: 10.7554/eLife.21143 (PMC5092059; doi:10.7554/eLife.21143)
Supplement: Supplementary file 1. — The statistical data were obtained using Graphpad Prism 5. DOI: http://dx.doi.org/10.7554/eLife.21143.023 [file elife-21143-supp1.docx]

**Supplementary File 1**

Summary of statistics shown for the quantitative data in the Figures. The statistical data were obtained using Graphpad Prism 5.

**Fig. 1e Analysis of K_D_ for ^3^H gabapentin binding**

| Experiments | WT α_2_δ-1 K_D_ | V6 α_2_δ-1 K_D_ |
| --- | --- | --- |
| 1 | 70.69 | 67.56 |
| 2 | 88.72 | 89.28 |
| 3 | 88.89 | 61.06 |
| 4 | 56.68 | 122 |
| 5 | -- | -- |
| mean K_D_ | 76.245 | 84.975 |
| sem | 7.79513 | 13.7374 |

**Fig. 1g Analysis of α_2_(3C) δ-1 cell surface biotinylation, normalised to α_2_δ-1**

|  | normalised α_2_δ-1 | normalised  α_2_(3C) δ-1 |
| --- | --- | --- |
| Number of values | 4 | 4 |
|  |  |  |
| Minimum | 1.000 | 0.9109 |
| 25% Percentile | 1.000 | 0.9213 |
| Median | 1.000 | 1.016 |
| 75% Percentile | 1.000 | 1.157 |
| Maximum | 1.000 | 1.183 |
|  |  |  |
| Mean | 1.000 | 1.031 |
| Std. Deviation | 0.0 | 0.1236 |
| Std. Error | 0.0 | 0.06178 |
|  |  |  |
| Lower 95% CI of mean |  | 0.8346 |
| Upper 95% CI of mean |  | 1.228 |
|  |  |  |
| One sample t test |  |  |
| Theoretical mean |  | 1.000 |
| Actual mean |  | 1.031 |
| Discrepancy |  | -0.03124 |
| 95% CI of discrepancy |  | -0.1653 to 0.2278 |
| t, df |  | t=0.5057 df=3 |
| P value (two tailed) |  | 0.6479 |
| Significant (alpha=0.05)? |  | No |
|  |  |  |
| Sum | 4.000 | 4.125 |

**Fig. 1i Analysis of cell surface α_2_(3C) δ-1, normalised to α_2_δ-1, measured by immunocytochemistry**

| Table Analyzed | Data 1 |
| --- | --- |
| Column A | α_2_δ-1 WT |
| vs | vs |
| Column B | a2(3C)d1 |
| Unpaired t test |  |
| P value | 0.2948 |
| P value summary | ns |
| Are means signif. different? (P < 0.05) | No |
| One- or two-tailed P value? | Two-tailed |
| t, df | t=1.049 df=605 |
|  |  |
| How big is the difference? |  |
| Mean ± SEM of column A | 0.9931 ± 0.03535 N=290 |
| Mean ± SEM of column B | 0.9404 ± 0.03552 N=317 |
| Difference between means | 0.05266 ± 0.05022 |
| 95% confidence interval | -0.04577 to 0.1511 |
| R square | 0.001814 |
|  |  |
| F test to compare variances |  |
| F,DFn, Dfd | 1.104, 316, 289 |
| P value | 0.3935 |
| P value summary | ns |
| Are variances significantly different? | No |

**Fig. 2e Analysis of cell surface Ca_V_2.2-BBS, normalised to no α_2_δ, measured by immunocytochemistry**

| Table Analyzed | cell surface BBS |  |  |  |  |
| --- | --- | --- | --- | --- | --- |
|  |  |  |  |  |  |
|  |  |  |  |  |  |
| One-way analysis of variance |  |  |  |  |  |
| P value | < 0.0001 |  |  |  |  |
| P value summary | **** |  |  |  |  |
| Are means signif. different? (P < 0.05) | Yes |  |  |  |  |
| Number of groups | 3 |  |  |  |  |
| F | 37.62 |  |  |  |  |
| R square | 0.1157 |  |  |  |  |
|  |  |  |  |  |  |
| Bartlett's test for equal variances |  |  |  |  |  |
| Bartlett's statistic (corrected) | 77.64 |  |  |  |  |
| P value | < 0.0001 |  |  |  |  |
| P value summary | **** |  |  |  |  |
| Do the variances differ signif. (P < 0.05) | Yes |  |  |  |  |
|  |  |  |  |  |  |
| ANOVA Table | SS | df | MS |  |  |
| Treatment (between columns) | 234.0 | 2 | 117.0 |  |  |
| Residual (within columns) | 1788 | 575 | 3.110 |  |  |
| Total | 2022 | 577 |  |  |  |
|  |  |  |  |  |  |
| Dunnett's Multiple Comparison Test | Mean Diff. | q | Significant? P < 0.05? | Summary | 95% CI of diff |
| Cav2.2/B1b vs a2d1HA + CaV2.2/B1b | -1.412 | 7.970 | Yes | *** | -1.805 to -1.019 |
| Cav2.2/B1b vs α_2_δ-1(3C) + Cav2.2/B1b | -1.221 | 6.795 | Yes | *** | -1.619 to -0.8223 |
|  |  |  |  |  |  |

**Fig. 2f Analysis of resting membrane potential in the absence and presence of TASK3**

| Table Analyzed | Resting membrane potential (mV) |
| --- | --- |
| Column A | Control |
| vs | vs |
| Column B | TASK3 |
|  |  |
| Unpaired t test |  |
| P value | < 0.0001 |
| P value summary | **** |
| Are means signif. different? (P < 0.05) | Yes |
| One- or two-tailed P value? | Two-tailed |
| t, df | t=9.853 df=26 |
| How big is the difference? |  |
| Mean ± SEM of column A | -28.69 ± 2.891 N=16 |
| Mean ± SEM of column B | -70.17 ± 2.955 N=12 |
| Difference between means | 41.47 ± 4.209 |
| 95% confidence interval | 32.82 to 50.13 |
| R square | 0.7888 |
|  |  |
| F test to compare variances |  |
| F,DFn, Dfd | 1.276, 15, 11 |
| P value | 0.6937 |
| P value summary | ns |
| Are variances significantly different? | No |
|  |  |

**Fig. 2g Analysis of cell surface Ca_V_2.2-BBS in TASK3 transfected cells, normalised to no α_2_δ, measured by immunocytochemistry**

| Table Analyzed | Cell surface BBS |  |  |  |  |
| --- | --- | --- | --- | --- | --- |
|  |  |  |  |  |  |
| One-way analysis of variance |  |  |  |  |  |
| P value | < 0.0001 |  |  |  |  |
| P value summary | **** |  |  |  |  |
| Are means signif. different? (P < 0.05) | Yes |  |  |  |  |
| Number of groups | 3 |  |  |  |  |
| F | 11.02 |  |  |  |  |
| R square | 0.09070 |  |  |  |  |
| Bartlett's test for equal variances |  |  |  |  |  |
| Bartlett's statistic (corrected) | 98.28 |  |  |  |  |
| P value | < 0.0001 |  |  |  |  |
| P value summary | **** |  |  |  |  |
| Do the variances differ signif. (P < 0.05) | Yes |  |  |  |  |
|  |  |  |  |  |  |
| ANOVA Table | SS | df | MS |  |  |
| Treatment (between columns) | 118.6 | 2 | 59.32 |  |  |
| Residual (within columns) | 1189 | 221 | 5.382 |  |  |
| Total | 1308 | 223 |  |  |  |
| Bonferroni's Multiple Comparison Test | Mean Diff. | t | Significant? P < 0.05? | Summary | 95% CI of diff |
| No a2d vs + a2d1HA | -1.643 | 4.233 | Yes | **** | -2.519 to -0.7668 |
| No a2d vs + a2d13C | -1.494 | 3.946 | Yes | *** | -2.349 to -0.6397 |
|  |  |  |  |  |  |
| **Fig. 3e Analysis of time constant of activation for Ca_V_2.2 currents for the 4 conditions shown** | | | | | |
| Number of groups | 4 |  |  |  |  |
| F | 5.507 |  |  |  |  |
| R square | 0.1846 |  |  |  |  |
|  |  |  |  |  |  |
| Bartlett's test for equal variances |  |  |  |  |  |
| Bartlett's statistic (corrected) | 11.79 |  |  |  |  |
| P value | 0.0081 |  |  |  |  |
| P value summary | ** |  |  |  |  |
| Do the variances differ signif. (P < 0.05) | Yes |  |  |  |  |
|  |  |  |  |  |  |
| ANOVA Table | SS | df | MS |  |  |
| Treatment (between columns) | 13.61 | 3 | 4.537 |  |  |
| Residual (within columns) | 60.14 | 73 | 0.8239 |  |  |
| Total | 73.75 | 76 |  |  |  |
|  |  |  |  |  |  |
| Bonferroni's Multiple Comparison Test | Mean Diff. | t | Significant? P < 0.05? | Summary | 95% CI of diff |
| WT α_2_δ-1 vs no α_2_δ | -0.8694 | 2.870 | Yes | * | -1.563 to -0.1760 |
| α_2_δ-1(3C) vs α_2_δ-1(3C) + 3Cprotease | 0.7749 | 2.617 | Yes | * | 0.09713 to 1.453 |

**Fig. 3g Analysis of cell surface Ca_V_2.2-BBS, normalised to no α_2_δ, measured by immunocytochemistry for the 5 conditions shown.**

| Table Analyzed | SurfBBS |  |  |  |  |
| --- | --- | --- | --- | --- | --- |
|  |  |  |  |  |  |
| One-way analysis of variance |  |  |  |  |  |
| P value | < 0.0001 |  |  |  |  |
| P value summary | **** |  |  |  |  |
| Are means signif. different? (P < 0.05) | Yes |  |  |  |  |
| Number of groups | 5 |  |  |  |  |
| F | 20.50 |  |  |  |  |
| R square | 0.07678 |  |  |  |  |
|  |  |  |  |  |  |
| Bartlett's test for equal variances |  |  |  |  |  |
| Bartlett's statistic (corrected) | 97.70 |  |  |  |  |
| P value | < 0.0001 |  |  |  |  |
| P value summary | **** |  |  |  |  |
| Do the variances differ signif. (P < 0.05) | Yes |  |  |  |  |
|  |  |  |  |  |  |
| ANOVA Table | SS | df | MS |  |  |
| Treatment (between columns) | 304.1 | 4 | 76.03 |  |  |
| Residual (within columns) | 3657 | 986 | 3.709 |  |  |
| Total | 3961 | 990 |  |  |  |
|  |  |  |  |  |  |
| Bonferroni's Multiple Comparison Test | Mean Diff. | t | Significant? P < 0.05? | Summary | 95% CI of diff |
| α_2_δ-1 + Cav2.2/B1b vs α_2_δ-1 + Cav2.2/B1b + 3C protease | -0.1980 | 1.032 | No | ns | -0.6781 to 0.2821 |
| a2d1 + Cav2.2/B1b vs Cav2.2/B1b | 1.362 | 7.227 | Yes | **** | 0.8901 to 1.833 |
| α_2_δ-1(3C) + Cav2.2/B1b vs α_2_δ-1(3C) + Cav2.2/B1b + 3C protease | 0.1232 | 0.6236 | No | ns | -0.3712 to 0.6176 |
| α_2_δ-1(3C) + Cav2.2/B1b vs Cav2.2/B1b | 1.231 | 6.275 | Yes | **** | 0.7403 to 1.722 |

**Fig. 4b Analysis of cell surface Ca_V_2.2-BBS in the presence of α_2_δ-3, normalised to no α_2_δ, measured by immunocytochemistry**

| Table Analyzed | Cell surface BBS |
| --- | --- |
| Column A | a2d3 + Cav2.2/B1b |
| vs | vs |
| Column E | Cav2.2/B1b |
|  |  |
| Unpaired t test |  |
| P value | 0.0028 |
| P value summary | ** |
| Are means signif. different? (P < 0.05) | Yes |
| One- or two-tailed P value? | Two-tailed |
| t, df | t=3.008 df=374 |
|  |  |
| How big is the difference? |  |
| Mean ± SEM of column A | 1.313 ± 0.08017 N=188 |
| Mean ± SEM of column E | 1.004 ± 0.06407 N=188 |
| Difference between means | 0.3087 ± 0.1026 |
| 95% confidence interval | 0.1076 to 0.5099 |
| R square | 0.02362 |
|  |  |
| F test to compare variances |  |
| F,DFn, Dfd | 1.565, 187, 187 |
| P value | 0.0023 |
| P value summary | ** |
| Are variances significantly different? | Yes |

**Fig. 4e Analysis of cell surface α_2_δ-3 (WT or 3C) in the presence of 3C protease, normalised to the value in the presence of inactive (C147V) protease**

|  | WT α_2_δ-3 +C147V | | WT α_2_δ-3 +3C | |
| --- | --- | --- | --- | --- |
| Number of values | 3 | | 3 | |
|  |  | |  | |
| Minimum | 1.000 | | 0.6143 | |
| 25% Percentile | 1.000 | | 0.6143 | |
| Median | 1.000 | | 0.7795 | |
| 75% Percentile | 1.000 | | 1.171 | |
| Maximum | 1.000 | | 1.171 | |
|  |  | |  | |
| Mean | 1.000 | | 0.8549 | |
| Std. Deviation | 0.0 | | 0.2859 | |
| Std. Error | 0.0 | | 0.1651 | |
|  |  | |  | |
| Lower 95% CI of mean | 1.000 | | 0.1447 | |
| Upper 95% CI of mean | 1.000 | | 1.565 | |
|  |  | |  | |
| One sample t test |  | |  | |
| Theoretical mean |  | | 1.000 | |
| Actual mean |  | | 0.8549 | |
| Discrepancy |  | | 0.1451 | |
| 95% CI of discrepancy |  | | -0.8553 to 0.5652 | |
| t, df |  | | t=0.8790 df=2 | |
| P value (two tailed) |  | | 0.4721 | |
| Significant (alpha=0.05)? |  | | No | |
|  |  | |  | |
| Sum | 3.000 | | 2.565 | |
|  | α_2_δ-1(3C) +C147V | α_2_δ-1(3C)+3C protease | |  |
| Number of values | 3 | 3 | |  |
|  |  |  | |  |
| Minimum | 1.000 | 0.7931 | |  |
| 25% Percentile | 1.000 | 0.7931 | |  |
| Median | 1.000 | 1.080 | |  |
| 75% Percentile | 1.000 | 1.106 | |  |
| Maximum | 1.000 | 1.106 | |  |
|  |  |  | |  |
| Mean | 1.000 | 0.9931 | |  |
| Std. Deviation | 0.0 | 0.1736 | |  |
| Std. Error | 0.0 | 0.1003 | |  |
|  |  |  | |  |
| Lower 95% CI of mean | 1.000 | 0.5617 | |  |
| Upper 95% CI of mean | 1.000 | 1.424 | |  |
|  |  |  | |  |
| One sample t test |  |  | |  |
| Theoretical mean |  | 1.000 | |  |
| Actual mean |  | 0.9931 | |  |
| Discrepancy |  | 0.006913 | |  |
| 95% CI of discrepancy |  | -0.4383 to 0.4245 | |  |
| t, df |  | t=0.06896 df=2 | |  |
| P value (two tailed) |  | 0.9513 | |  |
| Significant (alpha=0.05)? |  | No | |  |
|  |  |  | |  |
| Sum | 3.000 | 2.979 | |  |

**Fig. 5d Analysis of cell surface biotinylated/total α_2_(Th)δ-3 in the presence of extracellular thrombin, normalised to the value in the absence of thrombin**

|  | no Thrombin | +Thrombin |
| --- | --- | --- |
| Number of values | 4 | 4 |
|  |  |  |
| Minimum | 1.000 | 0.5721 |
| 25% Percentile | 1.000 | 0.6340 |
| Median | 1.000 | 0.8273 |
| 75% Percentile | 1.000 | 1.030 |
| Maximum | 1.000 | 1.095 |
|  |  |  |
| Mean | 1.000 | 0.8304 |
| Std. Deviation | 0.0 | 0.2136 |
| Std. Error | 0.0 | 0.1068 |
|  |  |  |
| Lower 95% CI of mean | 1.000 | 0.4905 |
| Upper 95% CI of mean | 1.000 | 1.170 |
|  |  |  |
| One sample t test |  |  |
| Theoretical mean |  | 1.000 |
| Actual mean |  | 0.8304 |
| Discrepancy |  | 0.1696 |
| 95% CI of discrepancy |  | -0.5094 to 0.1702 |
| t, df |  | t=1.588 df=3 |
| P value (two tailed) |  | 0.2105 |
| Significant (alpha=0.05)? |  | No |
|  |  |  |
| Sum | 4.000 | 3.322 |

**Fig. 5e Analysis of Ca_V_2.2 current amplitude in the 6 conditions shown**

| Kruskal-Wallis test |  |  |  |
| --- | --- | --- | --- |
| P value | < 0.0001 |  |  |
| Exact or approximate P value? | Gaussian Approximation |  |  |
| P value summary | **** |  |  |
| Do the medians vary signif. (P < 0.05) | Yes |  |  |
| Number of groups | 6 |  |  |
| Kruskal-Wallis statistic | 42.88 |  |  |
|  |  |  |  |
| Dunn's Multiple Comparison Test | Difference in rank sum | Significant? P < 0.05? | Summary |
| a2d3 vs a2d3+thrombin | 0.3750 | No | ns |
| a2d3-Thr vs a2d3-Thr+thrombin | 21.12 | Yes | * |
| NO a2d vs NO a2d + thrombin | -1.779 | No | ns |

| Table Analyzed | Data 1 |
| --- | --- |
| Column C | norm control for a2(3c)d1 |
| vs | vs |
| Column E | norm a2(3c)d1 |
|  |  |
| Unpaired t test |  |
| P value | 0.0067 |
| P value summary | ** |
| Are means signif. different? (P < 0.05) | Yes |
| One- or two-tailed P value? | Two-tailed |
| t, df | t=2.968 df=24 |
|  |  |
| How big is the difference? |  |
| Mean ± SEM of column C | 0.9999 ± 0.1245 N=12 |
| Mean ± SEM of column E | 0.5684 ± 0.08226 N=14 |
| Difference between means | 0.4315 ± 0.1454 |
| 95% confidence interval | 0.1314 to 0.7316 |
| R square | 0.2685 |
|  |  |
| F test to compare variances |  |
| F,DFn, Dfd | 1.963, 11, 13 |
| P value | 0.2475 |
| P value summary | ns |
| Are variances significantly different? | No |
|  |  |
|  |  |
| Table Analyzed | Data 1 |
| Column J | norm control for a2d1 |
| vs | vs |
| Column L | norm a2d1 |
|  |  |
| Unpaired t test |  |
| P value | 0.0486 |
| P value summary | * |
| Are means signif. different? (P < 0.05) | Yes |
| One- or two-tailed P value? | Two-tailed |
| t, df | t=1.994 df=107 |
|  |  |
| How big is the difference? |  |
| Mean ± SEM of column J | 1.000 ± 0.1022 N=55 |
| Mean ± SEM of column L | 1.305 ± 0.1142 N=54 |
| Difference between means | -0.3055 ± 0.1532 |
| 95% confidence interval | -0.6094 to -0.001554 |
| R square | 0.03584 |
|  |  |
| F test to compare variances |  |
| F,DFn, Dfd | 1.225, 53, 54 |
| P value | 0.4590 |
| P value summary | ns |
| Are variances significantly different? | No |

**Fig. 6d Analysis of effect of transfection of WT α_2_δ-1 or α_2_(3C)δ-1 on I_Ba_ in DRGs**

**Fig. 6f Analysis of cell surface expression of Ca_V_2.2-HA in the presence of no α_2_δ-1, WT α_2_δ-1 or α_2_(3C)δ-1**

| Table Analyzed | Data 1 |  |  |  |  |
| --- | --- | --- | --- | --- | --- |
|  |  |  |  |  |  |
| One-way analysis of variance |  |  |  |  |  |
| P value | < 0.0001 |  |  |  |  |
| P value summary | **** |  |  |  |  |
| Are means signif. different? (P < 0.05) | Yes |  |  |  |  |
| Number of groups | 3 |  |  |  |  |
| F | 15.97 |  |  |  |  |
| R square | 0.07965 |  |  |  |  |
|  |  |  |  |  |  |
| Bartlett's test for equal variances |  |  |  |  |  |
| Bartlett's statistic (corrected) | 56.83 |  |  |  |  |
| P value | < 0.0001 |  |  |  |  |
| P value summary | **** |  |  |  |  |
| Do the variances differ signif. (P < 0.05) | Yes |  |  |  |  |
|  |  |  |  |  |  |
| ANOVA Table | SS | df | MS |  |  |
| Treatment (between columns) | 13.47 | 2 | 6.734 |  |  |
| Residual (within columns) | 155.6 | 369 | 0.4217 |  |  |
| Total | 169.1 | 371 |  |  |  |
|  |  |  |  |  |  |
| Bonferroni's Multiple Comparison Test | Mean Diff. | t | Significant? P < 0.05? | Summary | 95% CI of diff |
| wt α_2_δ-1 vs α_2_(3C)δ-1 | 0.3812 | 4.981 | Yes | **** | 0.2090 to 0.5535 |
| wt α_2_δ-1 vs no α_2_δ | 0.4231 | 4.641 | Yes | **** | 0.2179 to 0.6284 |

**Fig. 7b Analysis of Ca_V_2.2-HA in processes, in presence of no α_2_δ-1, WT α_2_δ-1 or α_2_(3C)δ-1**

| Table Analyzed | Data 2 |  |  |  |  |
| --- | --- | --- | --- | --- | --- |
|  |  |  |  |  |  |
| One-way analysis of variance |  |  |  |  |  |
| P value | < 0.0001 |  |  |  |  |
| P value summary | **** |  |  |  |  |
| Are means signif. different? (P < 0.05) | Yes |  |  |  |  |
| Number of groups | 3 |  |  |  |  |
| F | 151.8 |  |  |  |  |
| R square | 0.3167 |  |  |  |  |
|  |  |  |  |  |  |
| Bartlett's test for equal variances |  |  |  |  |  |
| Bartlett's statistic (corrected) | 1193 |  |  |  |  |
| P value | < 0.0001 |  |  |  |  |
| P value summary | **** |  |  |  |  |
| Do the variances differ signif. (P < 0.05) | Yes |  |  |  |  |
|  |  |  |  |  |  |
| ANOVA Table | SS | df | MS |  |  |
| Treatment (between columns) | 122.6 | 2 | 61.28 |  |  |
| Residual (within columns) | 264.4 | 655 | 0.4037 |  |  |
| Total | 387.0 | 657 |  |  |  |
|  |  |  |  |  |  |
| Bonferroni's Multiple Comparison Test | Mean Diff. | t | Significant? P < 0.05? | Summary | 95% CI of diff |
| no α_2_δ norm vs α_2_δ-1 norm | -0.8874 | 15.33 | Yes | **** | -1.017 to -0.7573 |
| α_2_δ-1 norm vs α_2_δ-1(3C) norm | 0.9269 | 14.56 | Yes | **** | 0.7839 to 1.070 |

**Fig. 7d Analysis of Ca_V_2.2-HA in processes, in presence of α_2_(3C)δ-1 without or with 3C protease**

| Table Analyzed | Data 1 |
| --- | --- |
| Column C | a2(3C)d1 |
| vs | vs |
| Column D | a2(3C)d1+3C protease |
|  |  |
| Unpaired t test |  |
| P value | < 0.0001 |
| P value summary | **** |
| Are means signif. different? (P < 0.05) | Yes |
| One- or two-tailed P value? | Two-tailed |
| t, df | t=16.01 df=376 |
|  |  |
| How big is the difference? |  |
| Mean ± SEM of column C | 0.09274 ± 0.004624 N=191 |
| Mean ± SEM of column D | 0.3365 ± 0.01464 N=187 |
| Difference between means | -0.2437 ± 0.01522 |
| 95% confidence interval | -0.2736 to -0.2139 |
| R square | 0.4054 |
|  |  |
| F test to compare variances |  |
| F,DFn, Dfd | 9.817, 186, 190 |
| P value | < 0.0001 |
| P value summary | **** |
| Are variances significantly different? | Yes |

**Fig. 7f Analysis of WT α_2_δ-1 and α_2_(3C)δ-1 in processes when expressed alone**

| Table Analyzed | Data 1 |
| --- | --- |
| Column A | a2d1-WT-HA |
| vs | vs |
| Column B | a2d1(3C)-HA |
| Unpaired t test |  |
| P value | 0.2849 |
| P value summary | ns |
| Are means signif. different? (P < 0.05) | No |
| One- or two-tailed P value? | Two-tailed |
| t, df | t=1.070 df=507 |
|  |  |
| How big is the difference? |  |
| Mean ± SEM of column A | 0.9786 ± 0.04219 N=246 |
| Mean ± SEM of column B | 1.045 ± 0.04524 N=263 |
| Difference between means | -0.06645 ± 0.06208 |
| 95% confidence interval | -0.1881 to 0.05522 |
| R square | 0.002255 |
|  |  |
| F test to compare variances |  |
| F,DFn, Dfd | 1.229, 262, 245 |
| P value | 0.1017 |
| P value summary | ns |
| Are variances significantly different? | No |

**Fig. 7h Analysis of WT α_2_δ-1 and α_2_(3C)δ-1 in processes when expressed with Ca_V_2.2 and β1b**

| Table Analyzed | Data 1 |
| --- | --- |
| Column A | WT |
| vs | vs |
| Column B | PS |
| Unpaired t test |  |
| P value | < 0.0001 |
| P value summary | **** |
| Are means signif. different? (P < 0.05) | Yes |
| One- or two-tailed P value? | Two-tailed |
| t, df | t=5.312 df=403 |
|  |  |
| How big is the difference? |  |
| Mean ± SEM of column A | 0.9850 ± 0.04803 N=221 |
| Mean ± SEM of column B | 0.6319 ± 0.04448 N=184 |
| Difference between means | 0.3530 ± 0.06647 |
| 95% confidence interval | 0.2228 to 0.4833 |
| R square | 0.06543 |
|  |  |
| F test to compare variances |  |
| F,DFn, Dfd | 1.401, 220, 183 |
| P value | 0.0184 |
| P value summary | * |
| Are variances significantly different? | Yes |

**Fig. 8c Analysis of GCaMP6f signal in absence or presence of co-transfected α_2_(3C)δ-1**

| Table Analyzed | Data 1 |
| --- | --- |
| Column A | control |
| vs | vs |
| Column B | a23Cd1 |
|  |  |
| Paired t test |  |
| P value | 0.0490 |
| P value summary | * |
| Are means signif. different? (P < 0.05) | Yes |
| One- or two-tailed P value? | Two-tailed |
| t, df | t=2.462 df=6 |
| Number of pairs | 7 |
|  |  |
| How big is the difference? |  |
| Mean of differences | 4.192 |
| 95% confidence interval | 0.02535 to 8.359 |
| R square | 0.5025 |
|  |  |
| How effective was the pairing? |  |
| Correlation coefficient (r) | 0.6822 |
| P Value (one tailed) | 0.0457 |
| P value summary | * |
| Was the pairing significantly effective? | Yes |

**Fig. 8e Analysis of GCaMP6f signal in presence of co-transfected α_2_(3C)δ-1 and absence or presence of 3C protease**

| Table Analyzed | Data 1 |
| --- | --- |
| Column A | 3C |
| vs | vs |
| Column B | 3C+Prot |
|  |  |
| Paired t test |  |
| P value | 0.0005 |
| P value summary | *** |
| Are means signif. different? (P < 0.05) | Yes |
| One- or two-tailed P value? | Two-tailed |
| t, df | t=6.141 df=7 |
| Number of pairs | 8 |
|  |  |
| How big is the difference? |  |
| Mean of differences | -4.177 |
| 95% confidence interval | -5.785 to -2.568 |
| R square | 0.8434 |
|  |  |
| How effective was the pairing? |  |
| Correlation coefficient (r) | 0.8621 |
| P Value (one tailed) | 0.0029 |
| P value summary | ** |
| Was the pairing significantly effective? | Yes |
